# Vascular endothelial growth factor A: friend or foe in the pathogenesis of HIV and SARS-CoV-2 infections?

**DOI:** 10.3389/fcimb.2024.1458195

**Published:** 2025-02-11

**Authors:** Mieke A. van der Mescht, Helen C. Steel, Ronald Anderson, Theresa M. Rossouw

**Affiliations:** Department of Immunology, Faculty of Health Sciences, University of Pretoria, Pretoria, South Africa

**Keywords:** chemokines, COVID-19, cytokines, HIV, SARS-CoV-2, VEGF

## Abstract

This review article discusses the role of vascular endothelial growth factor A (VEGF-A) in the pathogenesis of SARS-CoV-2 and HIV infection, both conditions being renowned for their impact on the vascular endothelium. The processes involved in vascular homeostasis and angiogenesis are reviewed briefly before exploring the interplay between hypoxia, VEGF-A, neuropilin-1 (NRP-1), and inflammatory pathways. We then focus on SARS-CoV-2 infection and show how the binding of the viral pathogen to the angiotensin-converting enzyme 2 receptor, as well as to NRP-1, leads to elevated levels of VEGF-A and consequences such as coagulation, vascular dysfunction, and inflammation. HIV infection augments angiogenesis via several mechanisms, most prominently, by the trans-activator of transcription (*tat*) protein mimicking VEGF-A by binding to its receptor, VEGFR-2, as well as upregulation of NRP-1, which enhances the interaction between VEGF-A and VEGFR-2. We propose that the elevated levels of VEGF-A observed during HIV/SARS-CoV-2 co-infection originate predominantly from activated immune cells due to the upregulation of HIF-1α by damaged endothelial cells. In this context, a few clinical trials have described a diminished requirement for oxygen therapy during anti-VEGF treatment of SARS-CoV-2 infection. The currently available anti-VEGF therapy strategies target the binding of VEGF-A to both VEGFR-1 and VEGFR-2. The blocking of both receptors could, however, lead to a negative outcome, inhibiting not only pathological, but also physiological angiogenesis. Based on the examination of published studies, this review suggests that treatment targeting selective inhibition of VEGFR-1 may be beneficial in the context of SARS-CoV-2 infection.

## 1 Introduction

At the outset of the COVID-19 pandemic, individuals presenting with underlying medical conditions and comorbidities were considered at higher risk for infection with SARS-CoV-2 and the development of complications (Gupta et al., 2023; Russell et al., 2023; Santra et al., 2023; Marušić et al., 2024). The comorbidities reported in the literature that increase risk include obesity, cancer, cardiovascular disease (ischemic heart disease, hypertension, coronary artery disease, congestive cardiac failure, and stroke) diabetes mellitus, obesity, respiratory diseases [chronic obstructive pulmonary disease (COPD), asthma, pulmonary hypertension, and cystic fibrosis], chronic kidney disease, and immunodeficiency (especially people living with HIV [PLWH]) (Gupta et al., 2023; Russell et al., 2023; Santra et al., 2023; Marušić et al., 2024). Interestingly, most investigations on PLWH co-infected with SARS-CoV-2 have reported mild-to-moderate disease with only a few studies finding increased severity and mortality in PLWH (Gatechompol et al., 2021). Risk factors for developing severe SARS-CoV-2 in virally suppressed PLWH are generally similar to HIV-uninfected individuals (Ho et al., 2021).

SARS-CoV-2 is considered a prothrombotic viral infection. Acute coronary syndrome, pulmonary embolism, and deep vein thrombosis are some of the main thromboembolic manifestations observed in patients with COVID-19 (Acherjee et al., 2021). A meta-analysis evaluating the incidence of thrombotic events in patients hospitalized with COVID-19, which included 20,886 patients from 43 studies, reported that venous thromboembolisms occurred in 7.9% (95% CI 5.1–11.2), deep vein thrombosis in 4.1% (95% CI 2.3–6.4%) and pulmonary embolism in 3.5% (95% CI 2.2–5.1%) of patients (Nopp et al., 2020). SARS-CoV-2-induced venous thromboembolism is caused by multiple mechanisms which include i) increased intracellular levels of von Willebrand factor which leads to impairment of endothelial function, ii) exacerbated systemic inflammation initiated by Toll-like receptor activation, and iii) the release of tissue factors by an activated intrinsic coagulation cascade (Acherjee et al., 2021). The inflammatory hyperresponsiveness, defined by increased C-reactive protein (CRP), interleukin (IL)-6, and fibrinogen concentrations, observed during COVID-19 causes an increase in D-dimer levels (Acherjee et al., 2021). Early studies that were done in Wuhan, China, reported a direct correlation between elevated IL-6 and a rise in D-dimer levels, indicating a link between inflammation and the procoagulant state observed during a SARS-CoV-2 infection (Acherjee et al., 2021). SARS-CoV-2 can also infect endothelial cells, resulting in the activation of transcription factors that initiate apoptosis, leading to the release of procoagulant factors (Acherjee et al., 2021). On admission to hospital with COVID-19, patients frequently present not only with coagulopathies but also with hypoxia (Acherjee et al., 2021). Hypoxia responses are orchestrated mainly by hypoxia-inducible factor (HIF).

In normoxic conditions, HIF subunit alpha 1(1α) is recognized by prolyl hydroxylase proteins (PHDs) and is consequently quickly degraded (with a half-life of approximately 5 minutes) by the von Hippel-Lindau tumor suppressor protein (VHL) (Sato and Takeda, 2023; Troise et al., 2023). The VHL protein regulates degradation of HIF-1α via the ubiquitin proteasome pathway by its association with cullin 2, elongins B and C, and ring-box 1 to form an E3 ubiquitin ligase complex that leads to the ubiquitination of HIF-1α (Troise et al., 2023). PHDs act as an oxygen sensitivity system for the HIF pathway before ubiquitination (Troise et al., 2023). During hypoxia, PHDs are inactivated, allowing HIF-1α and HIF-1β to migrate into the nucleus, dimerize, and bind to p300/CREB binding protein (CBP) to form a transcriptional activation complex that binds to the hypoxia response element (*HRE*) and activates the transcription of target genes (Sato and Takeda, 2023; Troise et al., 2023). Target genes that are upregulated during hypoxic conditions by HIF-1α are involved in multiple biological processes, such as i) angiogenesis [vascular endothelial growth factor A (VEGF-A) and angiopoietin 2 (*ANGPT2*)] (Marušić et al., 2024), ii) erythropoiesis [Erythropoietin (*EPO*)], iii) cell metabolism [Glucose Transporter 1 (*SLC2A1*), Hexokinase 1 (*HK1*) and Lactate Dehydrogenase A (*LDHA*)], iv) iron metabolism [Heme Oxygenase 1 (*HO1*) and transferrin (*TF*)], and v) apoptosis and cell survival [BCL2Interacting Protein 3 (*BNIP3*) and insulin-like growth factor-1(*IGF1*)] (Cheng et al., 2017; Sato and Takeda, 2023; Troise et al., 2023).

VEGF-A is one of the main cytokines associated with angiogenesis (Dabravolski et al., 2022). Notably, increased concentrations of VEGF have been observed in individuals presenting with SARS-CoV-2-associated acute respiratory distress syndrome (ARDS). As such, VEGF-A, together with other endothelial cell adhesion molecules, is considered a biomarker of disease severity, contributing to coagulation dysfunction (Chen L. et al., 2020). Similarly, elevated levels of VEGF-A have been documented in PLWH despite virally suppressive antiretroviral therapy (ART) (Ascherl et al., 1999). Thus, it has been suggested by a few studies that anti-VEGF therapy could be beneficial in treating severe COVID-19 and various clinical trials are still ongoing (Islam et al., 2020; Pang et al., 2021; Patel et al., 2021; Blumberg et al., 2022; Fanning et al., 2022; ClinicalTrials.gov, 2024a; ClinicalTrials.gov, 2024b; ClinicalTrials.gov, 2024c; ClinicalTrials.gov, 2024d; ClinicalTrials.gov, 2024e).

Van der Mescht et al. recently reported significantly elevated levels of VEGF in PLWH co-infected with SARS-CoV-2 compared to those infected with either HIV or SARS-CoV-2 alone (van der Mescht et al., 2023). Interestingly, increased VEGF concentrations were only observed in PLWH with virally suppressed HIV (van der Mescht et al., 2023). The findings of this study suggest that PLWH co-infected with SARS-CoV-2 present with less hypoxia than those without HIV, alluding to an attenuation of the association between hypoxia and elevated levels of VEGF in PLWH (van der Mescht et al., 2023).

In this review article, the role of VEGF-A in the pathogenesis of SARS-CoV-2 and HIV infection is explored, as well as the implications for therapeutic strategies.

## 2 Vascular homeostasis

**Figure 1:**
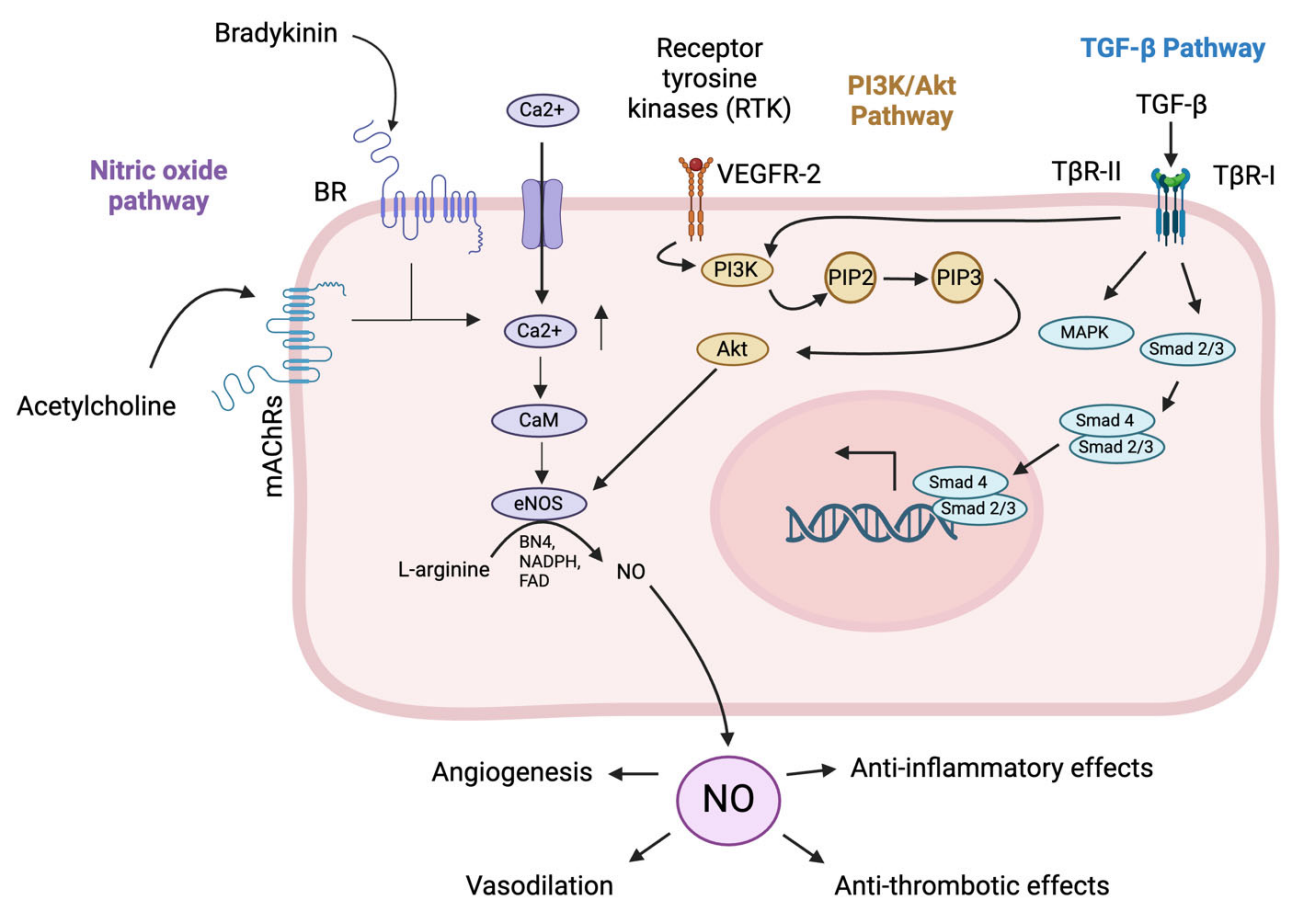
Pathways involved in endothelial cell homeostasis. The NO pathway cascade in endothelial cells begins with the activation of endothelial nitric oxide synthase (eNOS) by stimuli such as shear stress, hormones, and inflammatory cytokines (Durán et al., 2010). Activated eNOS converts L-arginine to nitric oxide (NO) and L-citrulline, requiring co-factors such as tetrahydrobiopterin (BH4) and calcium/calmodulin (Durán et al., 2010). NO then diffuses into adjacent vascular smooth muscle cells (VSMCs) and activates soluble guanylate cyclase (sGC), which converts guanosine triphosphate (GTP) to cyclic guanosine monophosphate (cGMP) (Durán et al., 2010). Increased cGMP levels activate protein kinase G (PKG), leading to the relaxation of VSMCs and vasodilation (Janaszak-Jasiecka et al., 2023). The functional outcomes include vasodilation, anti-thrombotic effects by inhibiting platelet aggregation, and anti-inflammatory effects by modulating adhesion molecule expression on endothelial cells (Durán et al., 2010; Janaszak-Jasiecka et al., 2023). The phosphatidylinositol 3-kinase (PI3K)/protein kinase B (Akt) pathway in endothelial cells begins with the binding of extracellular signals like growth factors such as vascular endothelial growth factor (VEGF) or insulin to receptor tyrosine kinases (RTKs) on the cell surface, leading to RTK autophosphorylation (Zhao et al., 2021). This creates docking sites for PI3K, which then phosphorylates phosphatidylinositol (3,4,5)-trisphosphate (PIP)2 to generate PIP3 (Zhao et al., 2021). PIP3 recruits Akt, with activated Akt in turn phosphorylating downstream targets, promoting cell survival by inactivating pro-apoptotic factors, enhancing protein synthesis via mechanistic Target Of Rapamycin (mTOR) activation, increasing glucose uptake, and boosting NO production for angiogenesis (Zhao et al., 2021). The pathway supports endothelial cell survival, growth, angiogenesis, and metabolic regulation, crucial for vascular integrity and function. The transforming growth factor beta (TGF-β) pathway in endothelial cells begins with the binding of TGF-β ligands to their receptors, transforming growth factor beta receptor (TβR)-II and TβR-I (Huang and Chen, 2012). Upon ligand binding, TβR-II phosphorylates and activates TβR-I, which activates Smad1/5/8, while activin receptor-like kinase 5 (ALK5) activates Smad2/3 (Huang and Chen, 2012). Smad’s form complexes with Smad4 and translocate to the nucleus to regulate gene transcription (Tzavlaki and Moustakas, 2020). Additionally, TGF-β can activate non-Smad pathways such as MAPK, PI3K/Akt, and Rho-like GTPase, influencing cell proliferation, survival, and migration (Huang and Chen, 2012). The functional outcomes include regulating endothelial cell growth and differentiation, maintaining vascular homeostasis, and promoting angiogenesis for wound healing and tissue regeneration. (Created in Biorender).

The vascular endothelium controls functions such as inflammation, vascular remodeling, cell adhesion, and coagulation (Kamtchum-Tatuene et al., 2019). Endothelial homeostasis is the balance and proper functioning of endothelial cells, which line blood vessels and are crucial for vascular health. This balance is maintained through various mechanisms, including the regulation of vascular tone via nitric oxide (NO) and endothelin, the selective barrier function controlling molecule passage, and the promotion of angiogenesis for new blood vessel formation (Incalza et al., 2018; Peng et al., 2019). Key pathways involved include the NO pathway for vasodilation, the Wnt/β-catenin pathway for cell regulation, the VEGF pathway for angiogenesis, and the TGF-β pathway for vascular integrity (**Figure 1**) (Walshe et al., 2009; van Meeteren and Ten Dijke, 2012; Tran et al., 2016; Janaszak-Jasiecka et al., 2023).

Different signals can influence the homeostasis of vascular endothelial cells (Chien, 2007). For instance, mechanical stimuli (resulting from circulatory pressure and flow) regulate vascular endothelial function by initiating mechanosensors and signaling pathways, as well as protein and gene expression (Chien, 2007). Mechanical stimuli with an explicit direction lead to transient molecular signaling of proliferative and pro-inflammatory pathways (Chien, 2007). On depletion or removal of these stimuli, the proliferative and pro-inflammatory pathways are downregulated (Chien, 2007). On the other hand, mechanical stimuli without an explicit direction can cause sustained signaling of these pathways, leading to vascular endothelial cell remodeling in order to reduce intracellular stress, as well as changes in vascular endothelial cell signaling to increase tolerance (Chien, 2007). An example of this is the initiation of glycolysis in vascular endothelial cells to promote angiogenesis and vessel sprouting (Lopaschuk et al., 2022). Another example is vascular endothelial cell proliferation secondary to the inhibition of mitochondrial respiration (Lopaschuk et al., 2022). Ultimately, all changes brought about by these mechanisms are balanced by feedback control mechanisms to preserve vascular homeostasis (Chien, 2007).

A healthy endothelium releases NO in response to thrombin secreted by aggregated platelets, which relaxes smooth muscle cells, increasing blood flow, and inhibiting coagulation (Vanhoutte et al., 2017). Shear stress also increases NO release through mechanisms such as bradykinin production in the human coronary circulation (Vanhoutte et al., 2017). The classical definition of endothelial dysfunction is the loss of NO production (Vanhoutte et al., 2017). A reduction of the amount of NO released by endothelial cells when the production of endothelial-derived contracting factor (EDCF) is enhanced is the hallmark of the initiation of endothelial dysfunction (Vanhoutte et al., 2017). The early phase of endothelial dysfunction involves cell activation, higher vascular permeability, and increased expression of adhesion molecules (Gimbrone and García-Cardeña, 2016). NO has a protective effect on the endothelium by preventing abnormal constriction (vasospasm) (Vanhoutte et al., 2017). It also inhibits the aggregation of platelets and the expression of adhesion molecules such as intercellular adhesion molecule 1 (ICAM-1), which brings about the adhesion and subsequent penetration of immune cells, as well as the release of vasoconstrictor and mitogenic peptide endothelin-1 (ET-1) (Vanhoutte et al., 2017). The abovementioned changes can be triggered by risk factors associated with cardiovascular disease such as hypertension, hyperlipidemia, insulin resistance, and hyperglycemia (Gimbrone and García-Cardeña, 2016). When endothelial dysfunction progresses into the late phase, a prothrombotic state ensues which can cause increased platelet production and subsequent platelet aggregation (Gimbrone and García-Cardeña, 2016). Aggregated platelets release powerful vasoconstrictors such as serotonin and thromboxane A2 (Vanhoutte et al., 2017). Consequent oxidative stress during this phase can also potentiate inflammation in the artery walls (Gimbrone and García-Cardeña, 2016). The aforementioned changes that occur during the late phase of endothelial dysfunction lead to endothelial cell senescence and death (Gimbrone and García-Cardeña, 2016).

The resting endothelium is equipped with cytokine receptors that coordinate particle clearance by resident macrophages (Flaumenhaft et al., 2022). The pulmonary capillaries account for approximately half of the vascular system in the body and are under continuous immune surveillance by neutrophils and macrophages (Flaumenhaft et al., 2022). If no infection is present, the pro-inflammatory response pathways, such as nuclear factor kappa light chain enhancer of activated B cells (NF-κB), myeloid differentiation primary response 88 (MyD88), signal transducer and activator of transcription 3 (STAT3), and mitogen-activated protein kinases (MAPK), are inhibited by cytoprotective receptors (Flaumenhaft et al., 2022).

## 3 Role of VEGF in angiogenesis

Steps involved in angiogenesis include enzymatic degradation of the capillary basement membrane, vascular endothelial cell proliferation, directed migration of vascular endothelial cells, tubulogenesis, vessel fusion, vessel pruning, and pericyte stabilization (Felmeden et al., 2003; Adair and Montani, 2010; Castro et al., 2018). Endothelial tip cells guide the maturing capillaries through the extracellular matrix (ECM) towards an angiogenic stimulus (Adair and Montani, 2010). Angiogenesis is initiated by angiotensin (Ang-2) and VEGF through the activation of the MAPK and protein kinase B (Akt) signaling pathways, which lead to the secretion of proteolytic enzymes (matrix metalloproteinases) by filopodia (Felmeden et al., 2003; De Smet et al., 2009; Adair and Montani, 2010; Barillari, 2020; Shi et al., 2023). Filopodia release substantial amounts of proteolytic enzymes that digest a pathway through the extracellular matrix for the development of the capillary sprout (De Smet et al., 2009; Adair and Montani, 2010; Castro et al., 2018). These enzymes cause the detachment of pericytes from vessels, cleave endothelial cell adhesion molecules, and degrade the basement membrane of the cell, thereby releasing the angiogenic molecules contained within the cells (Castro et al., 2018; Barillari, 2020).

VEGF undergoes alternative exon splicing, resulting in multiple isoforms, including VEGF_121_ (a highly diffusible isoform), VEGF_165_, VEGF_189_ (an ECM-bound isoform), and VEGF_206_ (Apte et al., 2019). The relative proportions of these VEGF-A isoforms vary across different tissues due to their specific roles in the later stages of vascular development and the affinity of various VEGF-A receptors (Mackenzie and Ruhrberg, 2012).

VEGF_165_, the most physiologically relevant isoform, is also the most highly expressed in tissues. It exhibits characteristics shared by VEGF_121_ and VEGF_189_, being partially matrix-bound and partially diffusible (Mackenzie and Ruhrberg, 2012; Apte et al., 2019). The diffusibility of these isoforms are linked to their varying affinity for heparan sulfate proteoglycans (HSPGs) in the ECM, although there is still a lack of genetic or physiological evidence supporting the hypothesis that HSPGs are essential for VEGF-A isoform-induced signaling events (Mackenzie and Ruhrberg, 2012).

VEGF_121_ has minimal affinity for heparin, whereas VEGF_189_ and VEGF_206_ each possess two heparin-binding domains encoded by exons 6 and 7, which anchor the protein to the cell surface or ECM (Apte et al., 2019). VEGF_165_ contains a single heparin-binding domain, encoded by exon 7, making it partially diffusible and partially ECM-bound (Apte et al., 2019). Proteolytic processing at the carboxyl terminus by enzymes such as matrix metalloproteinase-3 (MMP3) and plasmin can convert ECM-bound peptides into non-heparin-binding, diffusible molecules (Apte et al., 2019). The structures of two fragments of VEGF_165_ have been determined: the heparin-binding domain (HBD), which consists of 111–165 residues and is named VEGF_55_ and the receptor-binding domain (RBD), which has between 1 and 110 residues, and is termed VEGF_110_ (Ye et al., 2021). The structure of the full-length VEGF_165_ has yet to be determined (Ye et al., 2021).

Less common isoforms, such as VEGF_145_ and VEGF_183_, have been identified (Apte et al., 2019). A distinguishing feature of these isoforms is their varying ability to bind heparin (Apte et al., 2019). Recently, several inhibitory isoforms of VEGF, including VEGF_165b_ and VEGF-Ax, have been described (Apte et al., 2019). However, there is some controversy regarding their mechanisms of inhibition (Apte et al., 2019). Notably, VEGF-Ax has been shown to possess pro-angiogenic and pro-permeability properties (Apte et al., 2019).

VEGF-A plays an important role in angiogenesis and is secreted by many types of immune cells, such as macrophages, dendritic cells, activated T-cells and mast cells, and is also found in the α-granules of platelets (Gavalas et al., 2012; Morrell et al., 2014; Li et al., 2016; McHale et al., 2019; Domokos et al., 2023). VEGF-A has two receptors: VEGF receptor (VEGFR)-1 and VEGFR-2. VEGFR-1 has a higher binding affinity for VEGF-A, but the tyrosine kinase activity of this receptor is 10-fold weaker than that of VEGFR-2, implying that it functions as a control receptor during angiogenesis (Talotta, 2022). Because the tyrosine kinase activity of VEGFR-1 is so much weaker, VEGF-A is unable to stimulate the proliferation of cells overexpressing VEGFR-1 (Shibuya, 2006). Thus, VEGFR-1 can act as a positive regulator of angiogenesis by contributing a mild signal towards migration and proliferation (Shibuya, 2006). Indeed, it has been demonstrated *in vivo* that angiogenesis activated via VEGFR-1 is insignificant (Shibuya, 2006). On the other hand, VEGFR-1 can act as a negative regulator of angiogenesis by sequestering VEGF-A via its ligand-binding domain (Shibuya, 2006).

With respect to macrophage function, VEGF-A enhances the recruitment of these cells (Schnittman et al., 2023). VEGFR-1 is present on the surface of macrophages and monocytes and can cause an adverse immune response upon stimulation (Shibuya, 2006; Ohkubo et al., 2014; Varricchi et al., 2018; Talotta, 2022). The binding of VEGF-A to VEGFR-1 on the surface of monocytes induces migration and chemotaxis of cluster of differentiation (CD)16+ monocytes (Varricchi et al., 2018). VEGFR-1 is more abundantly expressed on the surface of CD16+ (intermediate and non-classical) monocytes than CD16- (classical) monocytes (Varricchi et al., 2018). In monocytes, signaling through VEGFR-1 activates the p38 kinase, ERK 1/2, MAPK, and PI3K/Akt pathways (Tchaikovski et al., 2008). Thus, activation of VEGFR-1 receptors on monocytes and macrophages can increase the secretion of VEGF-A in VEGF-stimulated monocyte-derived macrophages (Ohkubo et al., 2014; Corliss et al., 2016).

**Figure 2:**
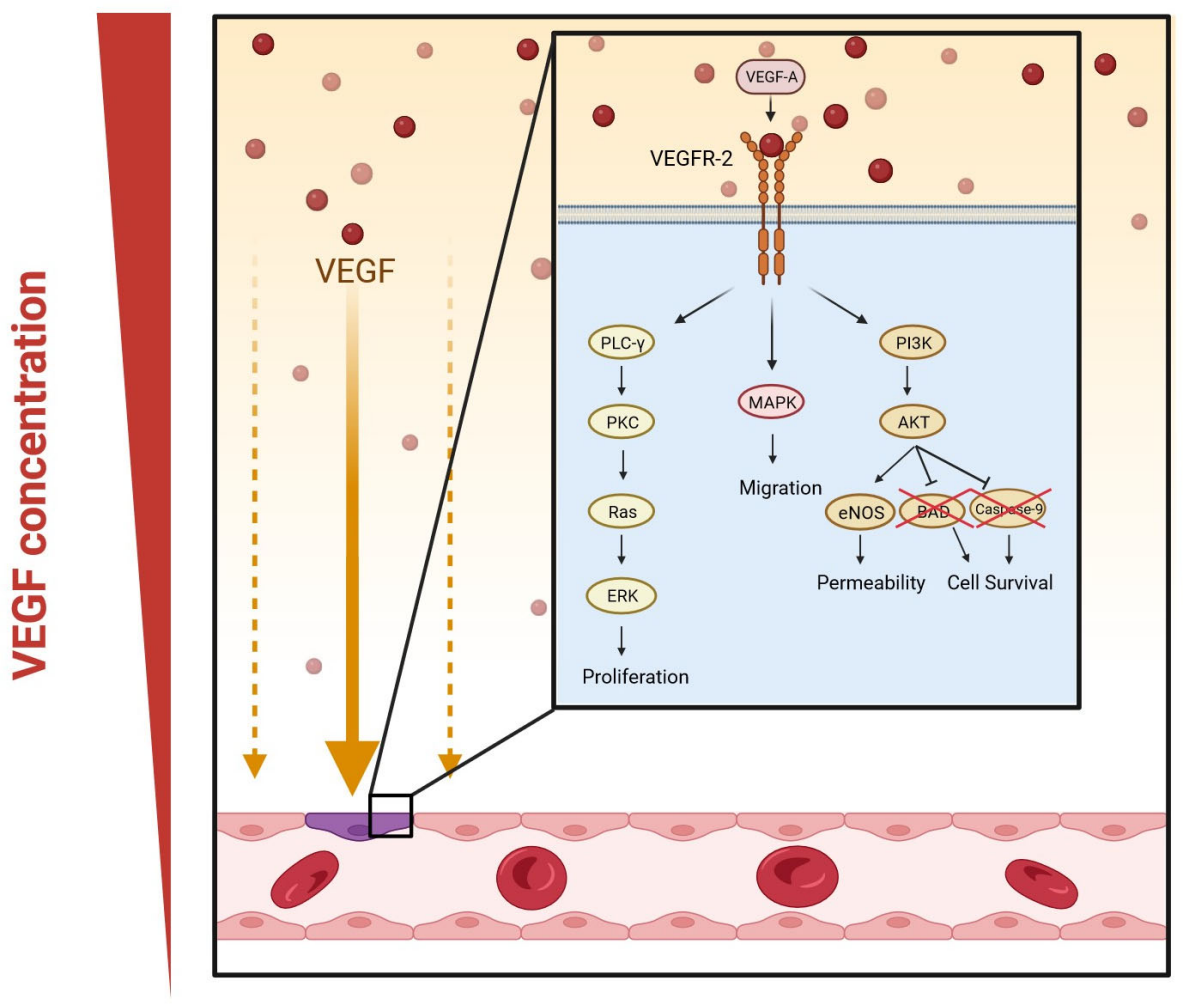
VEGF-A signaling pathways in angiogenesis. Dimerization occurs when VEGF-A binds to VEGFR-2. This activates multiple signaling pathways: phospholipase C gamma (PLC-γ) and protein kinase C (PKC), mitogen-activated protein kinases (MAPK), and phosphatidylinositol 3-kinase (PI3K)/protein kinase B (Akt) pathway. The PLC-γ/PKC pathway leads to the activation of extracellular signal-regulated kinase (ERK) and eventually the proliferation of vascular endothelial cells; MAPK increases migration; and the Akt pathway enhances the permeability and survival of the cells by increasing the expression of endothelial nitric oxide synthase (eNOS) and inhibiting Bcl-2-associated death promoter (BAD) and caspase-9. (Created in Biorender).

The most important complex in the VEGF signaling pathway is VEGF/VEGFR-2 (Jeong et al., 2021). It induces the activation of multiple intracellular signaling pathways, such as the phospholipase C gamma (PLC-γ), protein kinase C (PKC), MAPK, and Akt pathways by means of dimerization (**Figure 2**) (Barillari, 2020; Jeong et al., 2021; Talotta, 2022). The positive effects of VEGF-A on angiogenesis and the underlying mechanisms of these effects are summarized in **Table 1**. The filopodia of tip cells abundantly express VEGFR-2; this characteristic allows the cells to sense slight changes in VEGF-A concentrations and align themselves with the VEGF-A concentration gradient (Adair and Montani, 2010). Once enough filopodia on a tip cell have anchored to the substratum, actin filaments within the filopodia pull the tip cell along in the direction of the VEGF-A stimulus (Adair and Montani, 2010). At the same time, endothelial stalk cells proliferate, trailing behind the tip cell, resulting in elongation of the capillary sprout (Adair and Montani, 2010). Vacuoles arise and combine to form a lumen within the stalk cells to become the trunk of the new capillary (Adair and Montani, 2010). At the source of VEGF-A secretion, the tip cells of two or more capillary sprouts converge and fuse to form a continuous lumen, through which oxygen-rich blood can flow (Adair and Montani, 2010).

**Table 1:** Positive effects of VEGF on angiogenesis.

Effect of VEGF on angiogenesis	Mechanism	Reference
Proliferation of vascular endothelial cells	Binding of VEGF-A to VEGFR-2. Activates PLC-γ/PKC signaling pathway, leading to activation of ERK. Activates MAPK pathway, leading to activation of ERK.	(Barillari, 2020; Jeong et al., 2021)
Migration of endothelial cells	Binding of VEGF-A to VEGF-2. Activates the p38 MAPK signaling pathway. Leads to the activation of LIM kinase 1 (LMK1) and phosphorylation of ANXA1.	(Pin et al., 2012; Barillari, 2020)
Promotion of vasodilation/vascular permeability	Binding of VEGF-A to VEGF-2. Activates PLC-γ/PKC signaling pathway. Akt phosphorylation promotes the expression of eNOS, leading to the release of nitric oxide.	(Barillari, 2020; Wang et al., 2020)
Endothelial cell survival	Activation of the TSAd-Src-PI3K-PKB/Akt signaling pathway. Akt activation leads to the phosphorylation of BAD, caspase-9, and IκB kinases.	(Li et al., 2015; Wang et al., 2020)

Akt, protein kinase B; ANXA1, annexin A1; BAD, Bcl-2-associated death promoter; eNOS, endothelial nitric oxide synthase; ERK, extracellular signal-regulated kinase; IκB, inhibitor of nuclear factor-κB; MAPK, mitogen-activated protein kinases; PLC-γ, phospholipase C gamma; PI3K, phosphatidylinositol 3-kinase; PKC, protein kinase C; VEGF-A, vascular endothelial growth factor A; VEGFR-2, vascular endothelial growth factor receptor 2.

Another key signaling pathway in sprout formation is the Delta-Notch signaling pathway, which is a cell-to-cell signaling system in which cell-bound Delta-like 4 (Dll4) binds to its Notch receptor on the neighboring cell (Adair and Montani, 2010). Once VEGF binds to VEGFR-2, endothelial tip cells secrete Dll4, thus promoting connectivity between proliferating cells by activating the notch receptors on stalk cells (Adair and Montani, 2010; Barillari, 2020). The activation of Notch receptors inhibits the production of VEGFR-2 in stalk cells (Cheng et al., 2017). This is necessary to halt the migration of stalk cells (Adair and Montani, 2010).

Upon completion of angiogenesis, endothelial specialization and rearrangement of vessel connectivity occur (Ouarne et al., 2021). Endothelial cells stabilize the newly formed vessels by recruiting pericytes via the release of platelet-derived growth factor-BB (PDGF-BB) and mechanical signals such as shear stress (Adair and Montani, 2010; Barillari, 2020). These processes are essential for remodeling to occur, such that efficient vascular remodeling leads to the maturation and quiescence of vascular networks (Ouarne et al., 2021). Vascular quiescence occurs when the vascular network is stabilized and stops evolving. This process can be reversed by initiation of angiogenesis through VEGF-A signaling, triggered by different pathological (e.g., viral infections such as SARS-CoV-2) and physiological (e.g., pregnancy) scenarios (Ouarne et al., 2021). Angiopoietin-1 is responsible for the stabilization of endothelial cells and can inhibit vascular leakage (Wada et al., 2013). It has been shown that Ang-1, through the activation of the receptor tyrosine kinase, Tie2, can suppress the expression of genes related to inflammation and coagulation (Wada et al., 2013). Thus, the Ang-Tie2 ligand-receptor system is involved in the regulation and maturation of the endothelium (Wada et al., 2013).

ARDS is defined as a syndrome of acute onset, with bilateral diffuse infiltrates, leading to oxygenation impairment and non-cardiogenic respiratory failure (Vassiliou et al., 2020). The pathophysiology is characterized by alveolar and capillary endothelial damage (Vassiliou et al., 2020). Disruption of the endothelial barrier causes the displacement of macromolecules and fluid into the interstitial and pulmonary air spaces, leading to pulmonary edema (Vassiliou et al., 2020). Hyaline membrane formation in the alveolar walls promotes the release of protein-rich fluid and neutrophils into the alveolar space (Vassiliou et al., 2020). Transport of molecules can either occur transcellularly through endothelial cells or paracellularly via inter-endothelial junctions (Vassiliou et al., 2020). During infection, the endothelium becomes leaky and inflamed (Vassiliou et al., 2020). These changes occur to the microvascular endothelial structure to allow immune cells (innate and adaptive) to cross the barrier and reach the site of infection (Vassiliou et al., 2020). However, under overwhelming pathological conditions, this can lead to the development of ARDS. Critical host responses that occur during ARDS include inflammation (the release of pro- and anti-inflammatory cytokines, which can exacerbate organ damage through endothelial injury), coagulation, and fibrinolysis (Vassiliou et al., 2020). Elevated activation of coagulation in conjunction with dysfunction of the anti-coagulant mechanisms are contributors to, and consequences of, ongoing lung injury (Vassiliou et al., 2020). Once lung injury occurs, endothelial homeostasis is disrupted, initiating a cascade of vascular events, such as the activation of extravascular receptors that seal off the damage by inhibiting fibrinolysis and increasing coagulation (Vassiliou et al., 2020).

VEGF-A can also have a protective role in ARDS (Ricard et al., 2021). VEGF-A is present in the alveolar space in healthy individuals and helps to maintain alveolar function (Ricard et al., 2021). Primary human type 2 alveolar epithelial cells express VEGF-A, which under normal conditions acts as a mitogen and stimulant of the alveolar epithelium (Madureira and Soares, 2021). Polymorphisms associated with lower plasma levels of VEGF have been reported in patients with ARDS (Medford et al., 2005; Medford et al., 2009). In addition, VEGFR-2 blockers can cause alveolar apoptosis and emphysema in adults, suggesting that VEGF-A has a pneumotropic role (Madureira and Soares, 2021).

The development of a healthy vasculature depends on VEGF-A (Adair and Montani, 2010). If VEGF-A levels are reduced by 50% during embryonic development, this proves fatal due to consequent vascular defects (Adair and Montani, 2010). On the other hand, continuously elevated levels of VEGF-A, as seen in tumors due to the overproduction of tip cells, cause the formation of disorganized vasculature (Adair and Montani, 2010). Some of the pathophysiological conditions and the underlying mechanisms associated with VEGF are summarized in **Table 2**.

**Table 2:** Pathophysiological conditions associated with VEGF and the mechanisms underlying these effects.

Pathophysiology associated with VEGF	Mechanism	Reference
Inhibits neovascularization	Disrupts vascular smooth muscle cell (VSMC) function.	(Greenberg et al., 2008)
Vessel destabilization	Ablates coverage of nascent vascular sprouts.	(Greenberg et al., 2008)
Abnormal vessel maturation	Negative regulator of VSMCs.	(Greenberg et al., 2008)
Vascular permeability and edema	Disrupts vascular barrier function resulting in injury to ischemic tissue.	(Weis and Cheresh, 2005)
Proliferative retinopathy	Associated with intraocular neovascularization and vascular leakage.	(Miller et al., 2013)
Age-related macular degeneration	Increases vascular permeability and inflammation.	(Miller et al., 2013; Amadio et al., 2016)
Chronic skin inflammation: psoriasis, atopic dermatitis, hidradenitis suppurativa	Increases microvascular permeability and angiogenesis.	(Akhtar et al., 2018; Jones et al., 2018; van Aanhold et al., 2020; Lee et al., 2021)
Pathogenesis of rheumatoid arthritis	Increased levels of immunoreactive VEGF in the synovial fluid.	(Malemud, 2007)
Mediator of allograft rejection	Associated with trafficking of human T cells into skin allografts *in vivo* in the humanized severe combined immunodeficiency disease (SCID) mouse.	(Reinders et al., 2003)
Enhances blood-brain-barrier permeability	Elevated VEGF levels following focal cerebral ischemia.	(Argaw et al., 2009)
Angiogenesis associated with polycystic ovary syndrome and endometriosis	Induces angiogenesis.	(Reynolds et al., 2002; Chen et al., 2021)
Tumor invasiveness, vascular density, metastasis, and recurrence	Disrupts vascular barrier.	(Hicklin and Ellis, 2005; Weis and Cheresh, 2005)

## 4 The interplay between VEGF-A and neuropilin-1

Neuropilin-1 (NRP-1) is a receptor found on endothelial cells that engages in cell signaling pathways involved in cell migration (Dabravolski et al., 2022). If VEGF-A is secreted close to blood vessels, it upregulates the expression of NRP-1 on the cell surface, creating a positive feedback loop by increasing the endothelial sensitivity to VEGF-A (Talotta, 2022). NRP-1 is most abundantly expressed by mesenchymal stem cells, endothelial cells, and vascular smooth muscle cells (Mayi et al., 2021). Other cells that express NRP-1 are CD8+ T cells, regulatory T cells, macrophages, and neurons (Mayi et al., 2021). PDGF and arterial injury both upregulate the expression of NRP-1 (Mayi et al., 2021). Although NRP-1 induces angiogenesis without the involvement of VEGF-A, the VEGF pathway cannot function properly without NRP-1, underscoring the critical role of NRP-1 in the functioning of the vascular system (Mayi et al., 2021).

Different isoforms of NRP-1 can be produced due to alternative splicing (Talotta, 2022). Some of these isoforms are soluble and can function as decoy receptors (Talotta, 2022). A review by Roy et al. showed that _S12_NRP-1 acts as a decoy, which inhibits VEGF_165_ binding to NRP-1 since it contains the a and b domains, but lacks the transmembrane and cytosolic residues (Roy et al., 2017). The b1 coagulation factor domain of NRP-1 and NRP-2 contains a conserved cleft, which is best suited for interacting with a C-terminal arginine necessary for ligand binding (Parker et al., 2012). All VEGF family members contain a C-terminal arginine (Parker et al., 2012). While NRP-1 is the functional receptor for VEGF-A, NRP-2 is the functional receptor for VEGF-C (Parker et al., 2012). The b1 domain, which also interacts with furin-cleaved ligands that share a motif with arginine amino acid residues, is common in VEGF-A (Talotta, 2022).

NRP-1 forms a complex with VEGFR-2 in endothelial cells (Mayi et al., 2021; Talotta, 2022). NRP-1 and NRP-2 both act as co-receptors to amplify the binding of VEGF to VEGFR-2 in blood vessels (Talotta, 2022). NRP-1 has strong pro-inflammatory and pro-atherogenic properties by promoting leukocyte trafficking to sites of inflammation (Schnittman et al., 2023). In a murine model, NRP-1+ CD4+ T cells that are highly activated and secrete interferon (IFN)-γ and tumor necrosis factor (TNF)-α, showed increased migration to the aorta, where they were elevated in atherosclerotic plaques (Schnittman et al., 2023). In this context, it is interesting that NRP-1 expression is upregulated by oxidized low-density lipoprotein (LDL) in the mouse model (Schnittman et al., 2023).

Murine models have also revealed an association between upregulated NRP-1 expression and age (Schnittman et al., 2023). Upregulated NRP-1 expression in these models led to inhibition of anti-thrombotic and anti-inflammatory pathways, ultimately leading to platelet and macrophage activation and fibrosis (Schnittman et al., 2023). In a study done in humans, other clinical factors that were associated with elevated NRP-1 include a lower CD4+ T cell count, male sex, and a history of hepatitis C virus infection (Schnittman et al., 2023).

NRP-1 further acts as a signaling ligand for plexin, semaphorins, integrins, and various growth factors, such as PDGF, hepatocyte growth factor (HGF), and TGF-β1 (Mayi et al., 2021). PDGF mediates the hypoxia-induced promotion of proliferation and survival of endothelial cells by initiating receptor dimerization and phosphorylation, leading to the activation of multiple signaling pathways such as Akt/PKB, MAPK/ERK and JAK/STAT (Li et al., 2015; Zou et al., 2022). PDGF further contributes to cell survival by protecting cells against mitochondria-dependent apoptosis through the activation of the Akt/STAT3 signaling pathway (Li et al., 2015). HGF is a multifunctional growth factor involved in anti-inflammatory responses, angiogenesis, and cell proliferation and migration (Kaga et al., 2012). HGF can induce angiogenesis without inducing vascular permeability and inflammation (Kaga et al., 2012). These interactions underscore the contribution of NRP-1 to cell survival, migration, and proliferation (Talotta, 2022).

## 5 Role of VEGF-A during hypoxia and inflammation

**Figure 3:**
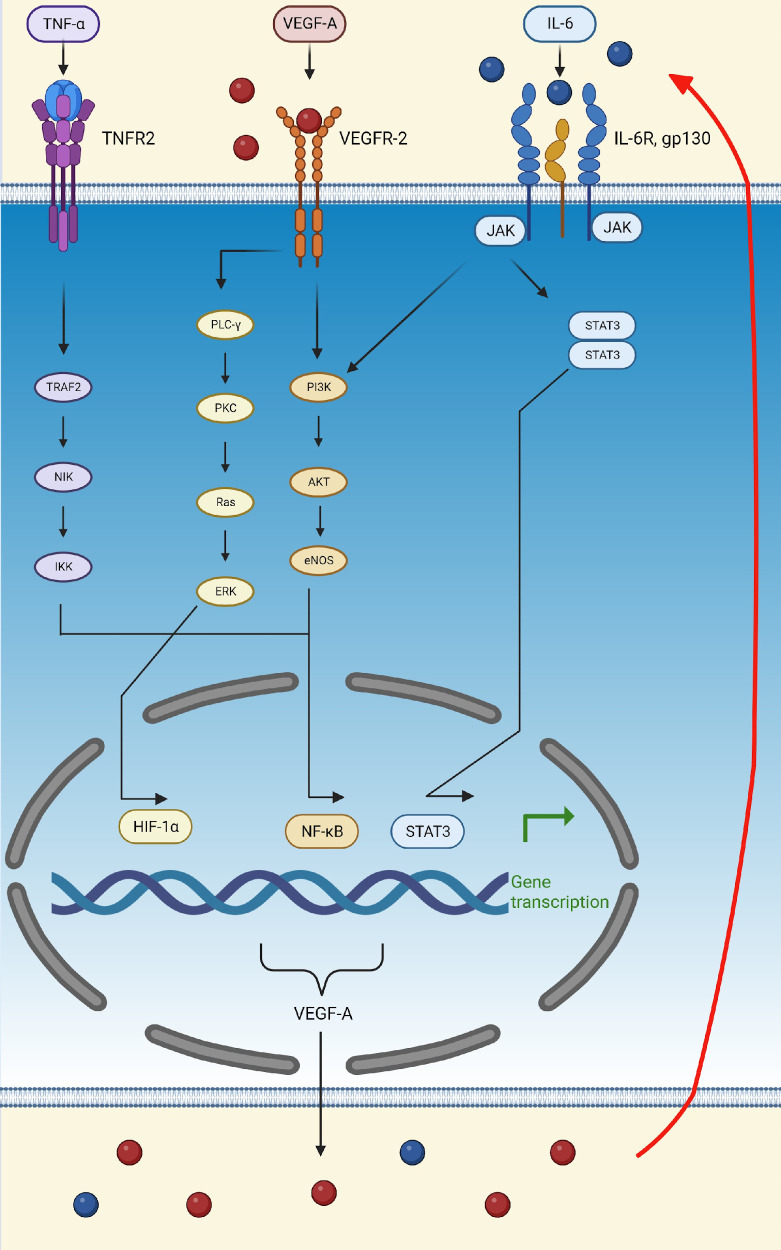
Schematic representation of the TNFα-NF-κB-HIF-VEGF pathway. TNF-α activates the NF-κB transcription factor by binding to TNFR2 and activating TRAF2, leading to the transcription of VEGF-A and other pro-inflammatory molecules, such as IL-6. VEGF-A can then attach to VEGFR-2 and activate the transcription factors, HIF-α and NF-κB, thus further upregulating the production of VEGF-A, IL-6, and TNF-α. Finally, IL-6 can bind to its receptor, IL-6R, activating the PI3K/Akt and STAT3 pathways. The activation of these pathways, initiated by VEGF-A, TNF-α, and IL-6, increases the production of VEGF-A, thus creating a positive feedback loop (indicated by the red arrow). (Created in Biorender). eNOS, endothelial nitric oxide synthase; ERK, extracellular signal-regulated kinase; Gp130, glycoprotein 130; HIF-1α, hypoxia-inducible factor 1 subunit alpha; IκB, inhibitor of nuclear factor-κB; IKK, kinase; IL-6, interleukin; IL-6R, interleukin 6 receptor; JAK, Janus kinase; NF-κB, nuclear factor kappa light chain enhancer of activated B cells; NIK, NF-κB induced kinase; PI3K, phosphatidylinositol 3-kinase; STAT3, signal transducer and activator of transcription 3; TNF-α, tumor necrosis factor-alpha; TNFR2, tumor necrosis factor receptor 2; TRAF2, TNF receptor-associated factor 2; VEGF-A, vascular endothelial growth factor A; VEGFR-2, vascular endothelial growth factor receptor 2.

During hypoxia and inflammation, endothelial cells are stimulated to release HIF-1α, which, via its role as a transcription factor, upregulates genes that mediate adaptive responses to lower oxygen availability, such as those encoding erythropoietin, VEGF-A, PDGF, and smooth muscle mitogen, to compensate for ischemic and hypoxic damage (Korgaonkar et al., 2008; Barillari, 2020; Dabravolski et al., 2022; Palakeel et al., 2022). VEGF-A is the only growth factor that can cause hypoxia-induced angiogenesis (Adair and Montani, 2010). This process is initiated in tissues during hypoxia in order to meet the metabolic needs of parenchymal cells (Adair and Montani, 2010). Under hypoxic conditions, endothelial cells also produce TNF-α, which activates the NF-κB pathway, thus creating the TNF-α-NF-κB-HIF-VEGF-A signaling cascade (Dabravolski et al., 2022). NF-κB activation is one of the most important events involved in triggering inflammation and proliferation (Dabravolski et al., 2022). In this context, VEGF-A can activate NF-κB through the Akt pathway (**Figure 3**) (Dabravolski et al., 2022).

In addition to TNF-α, lipopolysaccharide (LPS) and reactive oxygen species (ROS) also activate the TNFα-NF-κB-HIF-VEGF-A pathway, thereby upregulating IL-6, activating STAT3, and leading to the secretion of VEGF-A (Dabravolski et al., 2022). In turn, VEGF-A stimulates STAT3 in a positive feedback loop and amplifies its secretion (Dabravolski et al., 2022). Lastly, activation of the Akt pathway supports the feedback loop of the IL-6/STAT3 pathway (**Figure 3**) (Dabravolski et al., 2022).

Furthermore, VEGF-A can inhibit the inflammatory response, reduce oxidative stress, and promote the dilation and proliferation of lymphatic vessels (Dabravolski et al., 2022). In this context, VEGF-A can induce anti-inflammatory conditions by inhibiting the proliferation and function of cytotoxic T cells and through the recruitment of M2-type macrophages through the expression of C-X-C motif chemokine ligand 12 (CXCL12) (Elias et al., 2013; Li et al., 2015). Activated macrophages can be categorized into two distinct types: M1 macrophages, which play a role in promoting inflammatory responses, and M2 macrophages, which are associated with anti-inflammatory immune reactions (Yunna et al., 2020). High concentrations of CXCL12 are associated with some pathological conditions linked to hypoxia and a pro-angiogenic environment (Elias et al., 2013). VEGF-A alone only recruits macrophages, but cannot facilitate macrophage class switching from the M1 to the M2 phenotype (Elias et al., 2013). A study done by Shou et al. demonstrated *in vitro* that class switching from M1 to M2 phenotype macrophages is promoted by microRNA (miR)-126 via the downregulation of the VEGF-A/Krüppel-like factor 4 (KLF4) signaling pathway (Shou et al., 2023). KLF4 promotes cell proliferation and differentiation of monocytes, polarizing differentiation towards an M2 phenotype (Shou et al., 2023). In an M2-rich environment, the inflammatory response is downregulated, allowing tissue remodeling to occur (Shou et al., 2023).

Protein homeostasis is regulated by the endoplasmic reticulum (ER), which controls protein processing and folding (Wei et al., 2023). ER stress occurs when there is a disruption in the process of protein folding, which leads to an increase in the amount of misfolded proteins in the ER (Wei et al., 2023). HIF-1α is associated with ER stress and promotes apoptosis of alveolar epithelial cells (Luo et al., 2022; Wei et al., 2023). Endothelial cell survival is regulated by VEGF-A signaling under conditions of ER stress via the activation of unfolded protein response mediators through PLC-γ mediated cross-talk with the mechanistic Target Of Rapamycin (mTOR) kinase complex (Dabravolski et al., 2022). Other activated complexes that contribute to the survival effect of VEGF-A on endothelial cells are activating transcription factor 6 and protein kinase R-like ER kinase (Wei et al., 2023).

VEGF-A concentrations are downregulated, and angiogenesis terminates once the tissues receive adequate amounts of oxygen (Barillari, 2020; Talotta, 2022). This downregulation can occur via multiple mechanisms. The expression of VEGF is correlated with lipid levels, thus the TNFα-NF-κB-HIF-VEGF-A signaling cascade can be inhibited by LDL (Dabravolski et al., 2022). In this context, LDL suppresses TNF Receptor superfamily 1A and inhibits the expression of HIFs in endothelial cells, hence downregulating VEGF-A expression (Dabravolski et al., 2022). VEGF-A can decrease the activity of plasma lipoprotein lipase, which results in the accumulation of triglycerides in large protein granules and LDL (Dabravolski et al., 2022). Additionally, a cell cycle transcription factor, E2F Transcription Factor 1, can inhibit VEGF expression in a p53-dependent manner (Dabravolski et al., 2022), whereas placenta growth factor (PlGF) can inhibit VEGF-A in a p53-independent manner. Both events reduce the neovascularization and cardiac regeneration ability following injury (Dabravolski et al., 2022). During homeostatic angiogenesis, annexin A1 is a phospholipase A2 inhibitor with anti-inflammatory properties (Dabravolski et al., 2022). Thus, in addition to other mechanisms, the downregulation of annexin A1, which inhibits the activation of STAT3, further suppresses VEGF-A expression (Dabravolski et al., 2022).

As shown in **Table 2**, VEGF-A plays an important role in both physiological and pathological angiogenesis, the latter of which occurs during ischemic disease, microvascular occlusion, and inflammation (Dabravolski et al., 2022). Forms of pathological angiogenesis that are known to occur in the lungs include intussusceptive and sprouting angiogenesis, the latter occurring in asthma and chronic obstructive pulmonary disease, while intussusceptive angiogenesis and injured endothelium have been associated with SARS-CoV-2 infection (Jeong et al., 2021). Sprouting angiogenesis occurs when interconnected capillaries are formed from an existing vascular network (Ouarne et al., 2021). Intussusceptive angiogenesis, on the other hand, occurs when an intussusceptive pillar is formed, splitting and remodeling a blood vessel (Ouarne et al., 2021). VEGF-A signaling differs in sprouting and intussusceptive angiogenesis, depending on the local concentration of VEGF-A, as well as the location and the underlying pathological processes in lung tissue (Madureira and Soares, 2021). Lower concentrations of VEGF-A lead to intussusceptive angiogenesis, while higher concentrations of this growth factor lead to sprouting angiogenesis (Madureira and Soares, 2021). The transition between normal to abnormal angiogenesis depends on the concentrations and activities of PDGF-BB and VEGF-A (Meini et al., 2020). PDGF-BB recruits pericytes and thus acts as a regulator by stabilizing the vasculature, thereby preventing abnormal angiogenesis caused by the overexpression of VEGF-A, while a reduction in VEGF-A expression is associated with vascular tree regression by intussusceptive vascular pruning (Meini et al., 2020). Thus, the inhibition of VEGF-A is suggested to lead to vessel normalization by regulating intussusceptive angiogenesis (Meini et al., 2020).

## 6 VEGF-A upregulation in viral infections and its possible therapeutic implications

The NRP-1/VEGF-A axis has been implicated in the pathogenesis of several viral infections, such as those caused by human T cell lymphotropic virus type 1 (HTLV-1) and Epstein-Barr virus (EBV) (Talotta, 2022). The HTLV-1 envelope protein can mimic VEGF-A, promoting viral entry into cells expressing surface NRP-1 (Talotta, 2022). Knockdown of *NRP1* has been shown to suppress EBV infection while increasing *NRP1* expression has been shown to enhance EBV infection in human nasopharyngeal epithelial cells (Mayi et al., 2021). The two viral infections in which the NRP-1/VEGF-A axis has been implicated most strongly are, however, SARS-CoV-2 and HIV.

### 6.1 SARS-CoV-2 infection

SARS-CoV-2 is reported to enter cells in multiple ways, including via the angiotensin-converting enzyme 2 (ACE-2) receptor and NRP-1 (Ascherl et al., 1999; Shibuya, 2006; Cheng et al., 2017; Gatechompol et al., 2021; Talotta, 2022; Troise et al., 2023). SARS-CoV-2 has the highest affinity for entry via the ACE-2 receptor, which is expressed in vascular endothelial and respiratory cells, as well as on the surface of many types of immune cells, such as monocytes (Osman et al., 2021) and macrophages (Knoll et al., 2021), as well as in the heart (Romanowska-Kocejko et al., 2022), ileum (Burgueno et al., 2020), kidneys (Akpoviroro et al., 2024), bladder (Lin et al., 2021) and lungs (Li et al., 2020; Gatechompol et al., 2021; Rohlfing et al., 2021).

SARS-CoV-2 binds to the membrane-bound ACE-2 receptor to enter a cell. The strong binding affinity of SARS-CoV-2 to ACE-2 has made COVID-19 a greater threat than previous SARS viruses (Sodhi et al., 2022). This increased binding affinity is due to five amino acid changes (Asn501, Gln493, Leu455, Phe486, and Ser494), which lead to stronger hydrophobic interactions (Sodhi et al., 2022). Among these, Asn501 and Gln493 are the most crucial for van der Waals interactions and hydrogen bonding (Sodhi et al., 2022). The RBD of the viral spike (S) protein of SARS-CoV-2 interacts with the metallopeptidase domain of ACE-2, causing a structural change in the S protein that reveals cleavage sites at the S1/S2 regions (Sodhi et al., 2022).

Before entry, the S protein undergoes priming by the serine endopeptidase, transmembrane serine protease 2 (TMPRSS2), and the cysteine proteases, cathepsin B and L (CatB/L) (Sodhi et al., 2022). TMPRSS2 cleaves the S protein at subunit 1 and 2 sites and the S2 site, allowing the fusion of cellular and viral membranes (Sodhi et al., 2022). Once the S1 subunit binds to ACE-2, it promotes the cleavage of the ACE-2 ectodomain by the protease, A disintegrin and metalloproteinase 17 (ADAM-17), and the intracellular C-terminal domain by TMPRSS2, facilitating SARS-CoV-2 entry (Sodhi et al., 2022). ACE-2 is internalized along with the viral particles into endosomes (Sodhi et al., 2022).

The S protein not only gains entry through the ACE-2 receptor but also interferes with angiogenesis (Talotta, 2022). The ACE-2 receptor downregulates the expression of VEGF-A by preventing the phosphorylation of ERK-2 (Talotta, 2022). By occupying the binding site for the ACE-2 molecule, the S protein interferes with the control of ACE-2 over VEGF-A synthesis, thus increasing the levels of VEGF-A (Talotta, 2022).

Inflammation seen in COVID-19 starts once the virus invades alveolar lung epithelial cells (Silva et al., 2023). After infection, it leads to excessive activation of the ACE/Ang-2/angiotensin type 1 receptor (AT1R) axis, which, in turn, results in these cells being engulfed by antigen presenting cells, both activating and inducing innate and adaptive immune responses (Silva et al., 2023). The immune cells that are then recruited to the site of infection release large quantities of inflammatory mediators such as chemokine (C-C motif) ligand (CCL)7, CCL8, CCL13, CCL17, CD163, CXCL2, CXCL11, IFN-γ, IL-1, IL-2, IL-3, IL-4, IL-6, IL-7, IL-8, IL-9, IL-10, IL-13, IL-15, IL-17, interferon-inducible protein 10 (IP-10), macrophage inflammatory proteins (MIPs), regulated upon activation normal T-cell expressed and secreted (RANTES), soluble tumor necrosis factor receptor-1 (sTNFR1),TNF-α, TNF-β, and TNF-related apoptosis-inducing ligand (TRAIL) (Silva et al., 2023). The release of these numerous inflammatory mediators results in an explosive and uncontrolled immune response with severe symptoms and oxidative stress that has been coined as a cytokine storm (Silva et al., 2023). SARS-CoV-2 impacts both the pulmonary and systemic circulation through the renin-angiotensin system (RAS) pathway, leading to a prothrombotic state with hypercoagulability (Silva et al., 2023). Various immune and endothelial factors, including granzymes and VEGF, together with viral and host proteins such as caspases 6 and 8, intensify the inflammatory process (Silva et al., 2023). Monocytes and neutrophils contribute to the cytokine storm via inflammasome activation (Silva et al., 2023). An elevated neutrophil-to-lymphocyte ratio, activation of inflammasomes, and high levels of extracellular neutrophil traps (NETs) further exacerbate the inflammation (Silva et al., 2023). This ultimately leads to a hyperinflammatory state, which can be defined as an excessive and prolonged inflammatory response by the immune system. Clinical markers used to identify hyperinflammation include the neutrophil/lymphocyte ratio, serum D-dimer, ferritin, LDH and CRP levels (Silva et al., 2023).

The hyperinflammation caused by SARS-CoV-2 infection can upregulate the activation of the VEGF-A/VEGFR-2 pathway, initiating angiogenesis, NO production, vascular permeability, and disruption of endothelial cell junctions (Talotta, 2022). This, in turn, can contribute to interstitial edema and endothelial hyper-permeability (Ricard et al., 2021; Talotta, 2022). High levels of VEGF-A in SARS-CoV-2 infection result in the reorganization of vascular endothelial (VE)-cadherin away from junctional sites, via phosphorylation, leading to the internalization of the protein, thus causing vascular leakage (Flaumenhaft et al., 2022). VEGF-A also promotes vascular permeability and the production of Ang-2 and VE-tyrosine phosphatase, which synergize to block Tie2 signaling (Flaumenhaft et al., 2022). Once the Ang-2 levels exceed the Ang-1 levels, the endothelial barrier is destabilized by the activation of β1-integrins (Flaumenhaft et al., 2022). This, in turn, ultimately leads to the loss of barrier function in postcapillary venules, resulting in fluid extravasation (Flaumenhaft et al., 2022). SARS-CoV-2 can directly infect endothelial cells, causing cell lysis and death, further damaging the barrier integrity (Lambadiari et al., 2022). It has been proposed that deflated alveoli filled with plasma could be attractive to opportunistic bacteria and fungi (Cao, 2021). The cytokine-rich plasma also results in the recruitment of inflammatory cells (such as macrophages), further increasing lung inflammation (Cao, 2021). An increase in VEGF-A could also be a result of endothelial dysfunction, leading to local tissue hypoxia, which triggers a self-perpetuating mechanism (Talotta, 2022). Hypoxia-induced vascular leakage during SARS-CoV-2 infection plays a significant role in the pathogenesis of COVID-19 (Cao, 2021).

High VEGF-A plasma levels are reported during the early stages of SARS-CoV-2 infection (Madureira and Soares, 2021). A study by Josittus et al. that investigated whether VEGF-A levels varied between SARS-CoV-2-positive patients in terms of disease severity found that high VEGF-A levels measured on admission to hospital correlated with mortality (Josuttis et al., 2023). The study concluded that VEGF-A can be used to determine which patients are at risk of developing ARDS, acute kidney injury, or shock (Josuttis et al., 2023). In another study done by Rovas et al., the MYSTIC trial, which analyzed functional and biomarker data from 23 patients admitted to hospital with COVID-19, determined that circulating VEGF-A and ADAMTS13 levels, also known as von Willebrand factor-cleaving protease, as well as sublingual glycocalyx thickness were the main markers that were associated with disease severity and predictive of whether patients will develop ARDS (Rovas et al., 2021).

The elevated VEGF-A levels reported during SARS-CoV-2 infection are indicative of widespread microvascular injury (Talotta, 2022). It is, therefore, likely that high levels of VEGF-A in COVID-19 further increase alveolar damage (Madureira and Soares, 2021). Increased VEGF-A expression in patients with COVID-19 causes impaired pericyte function, which leads to intussusceptive angiogenesis in the lungs (Talotta, 2022). As VEGF-A levels increase, Ang-2 levels also rise. In combination, these two molecules lead to the formation of small perforations in the primordial capillary plexus, indicating intussusceptive angiogenesis (Madureira and Soares, 2021). Angiopoietins are involved in the cross-talk between endothelial cells and pericytes (Madureira and Soares, 2021). The secretion of Ang-2 by endothelial cells is stimulated by hypoxic conditions as well as TNF-α, thrombin, and turbulent blood flow (Madureira and Soares, 2021). Increased levels of Ang-2, due to downstream eNOS activity, cause lower NO availability (Lambadiari et al., 2022). This indicates that, although VEGF-A may play a role in the initiation of intussusceptive angiogenesis, angiopoietins are crucial for the subsequent phases (Madureira and Soares, 2021).

VEGF-A variations that emerge due to alternative splicing could also increase the concentrations of circulating pro-inflammatory cytokines, and chemokines such as IL-1β, IL-6, IL-8, IP-10, monocyte chemoattractant protein-1 (MCP-1), and TNF-α (Talotta, 2022). This contention is supported by the finding that, in animal models, overproduction of VEGF-A can cause inflammation, cardiac edema, and remodeling of myocyte interstitial spaces, which, in turn, lead to cardiac arrhythmias (Talotta, 2022). VEGF-A can also act on nociception, contributing to the neuropathy seen in SARS-CoV-2 infection (Talotta, 2022).

NRP-1 plays a multisystem role that can make it an ideal entry target for SARS-CoV-2 and could contribute to the multisystem impact of viral infection (Mayi et al., 2021). NRP-1 has two forms, a truncated secreted form and a transmembrane form; the latter interacting with SARS-CoV-2 (Mayi et al., 2021). The transmembrane form contains the binding site for growth factors such as VEGF-A (Mayi et al., 2021). If this binding site is occupied by a virus, the growth factors cannot bind to their receptors (Mayi et al., 2021). In order to establish if SARS-CoV-2 can use NRP-1 as an entry point to infect a cell, Cantuti-Castelvetri et al. generated lentiviral particles pseudo-typed with the SARS-CoV-2 S protein (Cantuti-Castelvetri et al., 2020). The group then proceeded to infect human embryonic kidney 293 T (HEK-293T) cells with the generated virus (Cantuti-Castelvetri et al., 2020). They found that NRP-1 expression alone did not promote infection; however, when co-expressed with ACE-2 and transmembrane protease serine 2, infection of HEK-293T cells was significantly enhanced (Cantuti-Castelvetri et al., 2020).

**Figure 4:**
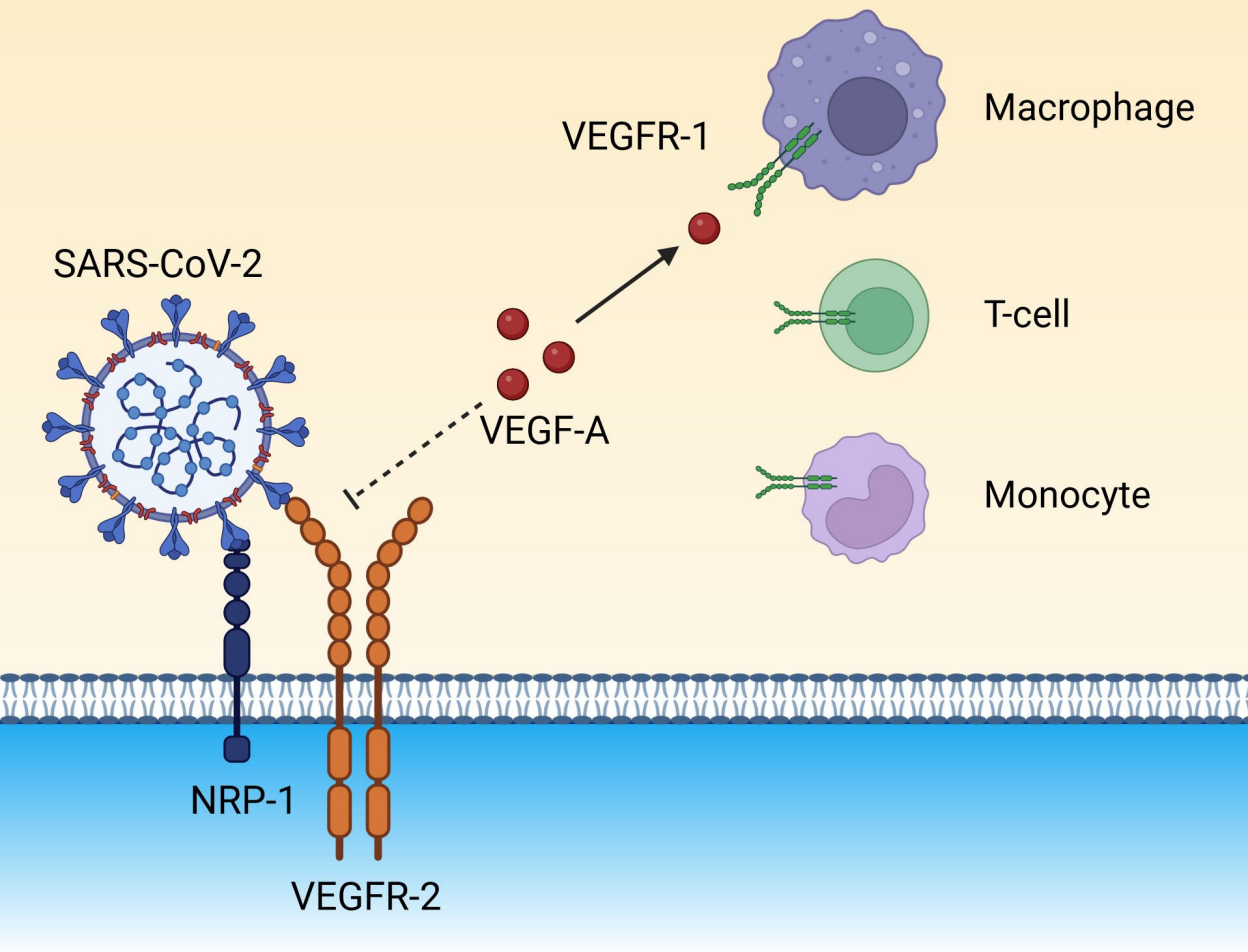
The spike protein on the surface of SARS-CoV-2 binds to NRP-1 on endothelial cells and destabilizes the NRP-1/VEGFR-2 receptor complex, eventually preventing the docking of the endogenous ligand, VEGF-A. Unbound VEGF-A could be diverted to VEGFR-1 expressed on immune cells (monocytes, macrophages, and T cells), amplifying the inflammatory background. (Created in Biorender). NRP-1, neuropilin-1; SARS-CoV-2, severe acute respiratory syndrome coronavirus 2; VEGF-A, vascular endothelial growth factor A; VEGFR-1, vascular endothelial growth factor receptor 1; VEGFR-2, vascular endothelial growth factor receptor 2.

SARS-CoV-2 can also be internalized via the NRP-1 pathway through the S1 protein by means of interaction with the b1 domain, which stimulates generalized intravascular coagulation and vascular dysfunction (Mayi et al., 2021; Talotta, 2022). As mentioned above, the b1 domain is also the binding site for VEGF-A (Mayi et al., 2021; Talotta, 2022). VEGFR-2 forms a bridge with NRP-1 via VEGF_165_, which binds to the b1 domain (Mayi et al., 2021). The S protein has a higher affinity for binding to NRP-1 than VEGF-A because the binding of S1 to NRP-1 cells destabilizes the NRP-1/VEGFR-2 complex, which displaces VEGF-A, contributing to the high serum concentrations of VEGF-A in patients with COVID-19 (Talotta, 2022). Free VEGF-A can then be diverted to bind with VEGFR-1 found on the surface of various types of immune cells (**Figure 4**) (Talotta, 2022).

Tissue expression of VEGFR-1 is elevated in patients with COVID-19 when compared to heathy control individuals and, interestingly, also when compared to patients with influenza A (Miggiolaro et al., 2023). The binding of VEGF-A to VEGFR-1 activates an inflammatory response, which further drives hyperinflammation (Talotta, 2022). High levels of circulating soluble VEGFR-1 have been linked to higher morbidity and mortality in ARDS (Wada et al., 2013). In circumstances in which levels of soluble VEGFR-1 are increased, it can sequestrate VEGF-A, consequently interfering with the involvement of VEGF-A in endothelium repair, which ultimately leads to disease progression and organ dysfunction (Wada et al., 2013; Ricard et al., 2021).

Severe COVID-19 infection also causes arterial damage, further increasing NRP-1 expression (Mayi et al., 2021), a contention supported by observations of increased NRP-1 RNA in cells infected with SARS-CoV-2 isolated from bronchoalveolar lavage fluid from patients with COVID-19 (Cantuti-Castelvetri et al., 2020). This increase was not seen in the uninfected cells from the same patients (Cantuti-Castelvetri et al., 2020).

Markers that indicate blood vessel damage and endothelial activation, such as Ang-2, ICAM-1, and IL-1β, are elevated in severe COVID-19 (Miggiolaro et al., 2023). In postcapillary venules, leukocyte recruitment is facilitated by the expression of adhesion molecules [such as vascular cell adhesion molecule 1 (VCAM-1) and ICAM-1] and selectins (e.g., E-selectin and P-selectin) on the pulmonary endothelium (Flaumenhaft et al., 2022). This combination causes severe vascular compromise, possibly accompanied by the development of microthrombi and the expression of inflammatory mediators, in patients with COVID-19 (Miggiolaro et al., 2023). The elevation of specifically ICAM-1 seen in COVID-19 indicates that SARS-CoV-2 is capable of significant endothelial damage (Miggiolaro et al., 2023).

An experimental animal infection study undertaken in Rhesus macaques to determine the mechanisms underlying the inflammation and thrombosis seen in SARS-CoV-2 infection, showed activation of IFN-α, IFN-γ, interleukins, and TNF-α in bronchoalveolar samples on the first day of infection (Flaumenhaft et al., 2022). These molecules lead to the recruitment of large numbers of macrophages and stimulate a pro-inflammatory response in the endothelium (Flaumenhaft et al., 2022). Similarly, Miggiolaro et al. demonstrated a significant increase in TNF-α and NF-κB expression in postmortem lung samples of patients who died of ARDS due to COVID-19 compared to the control group that died due to neoplastic or cardiovascular disease without the presence of lung lesions (Miggiolaro et al., 2023). They suggest that these results indicate a strong involvement of the TNF-α/TNFR1/NF-κB pathway in COVID-19 pathogenesis (**Figure 3**). As mentioned, the TNF-α/TNFR1/NF-κB pathway is activated during hypoxic conditions and can upregulate the expression of HIF-1α (Miggiolaro et al., 2023). In this context, it is noteworthy that increased expression of HIF-1α in lung vascular endothelial cells and bronchoalveolar lavage monocytes was observed in patients with COVID-19 (Codo et al., 2020; Miggiolaro et al., 2023). This mechanism also activates M1 macrophages, which, in turn, release multiple pro-inflammatory cytokines (Miggiolaro et al., 2023).

### 6.2 HIV infection

The other viral infection in which the NRP-1/VEGF-A axis has been implicated is HIV. HIV infects CD4+ T cells by attaching to the CD4 T cell receptor and its co-receptor CXCR4 or CCR5 (Masenga et al., 2023). The viral envelope glycoprotein (gp), gp120, first binds to the CD4 receptor on the T cell surface (Masenga et al., 2023). This interaction induces a conformational change in gp120, enabling it to subsequently bind to a co-receptor, either CXCR4 or CCR5 (Masenga et al., 2023). This interaction exposes the gp41, which then facilitates the fusion of the viral and cellular membranes (Masenga et al., 2023).

In healthy individuals, endothelial cells prevent platelet activation by expressing CD39 and CD73, which restrict the amount of available adenosine triphosphate and adenosine diphosphate and promote the release of the antiplatelet molecules, prostacyclin, and NO from endothelial cells (Perkins et al., 2023). During HIV infection, however, chronic inflammation leads to lower prostacyclin and NO production, as well as lower platelet inhibition secondary to the downregulation of CD39 and CD73 (Perkins et al., 2023). The extent of the ensuing endothelial dysfunction has been correlated with the level of HIV replication (Kuller et al., 2008).

Kamtchum-Tatuene et al. found that plasma levels of ICAM-1 were independently associated with HIV infection (Kamtchum-Tatuene et al., 2019). During homeostatic conditions, ICAM-1 is expressed on the surface of endothelial and epithelial cells at low levels. During an inflammatory response, ICAM-1 is upregulated and functions as a signaling receptor to convert outside-in signaling, linking leukocyte adhesive interactions to endothelial function (Bui et al., 2020). It also regulates intracellular calcium concentrations and endothelial cell permeability (Bui et al., 2020). Despite being on ART and having a stable infection (two consecutive undetectable viral load results and CD4+ T cell counts >200 cells/mm^3^) (Waldrop et al., 2016), PLWH still have ongoing endothelial activation (Kamtchum-Tatuene et al., 2019). High ICAM-1 levels have been associated with markers of atherosclerosis that could lead to cardiovascular events (Kamtchum-Tatuene et al., 2019). Cardiovascular disease (CVD), such as heart failure, atherosclerotic CVD, myocardial infarction, sudden cardiac death, arrythmias, and strokes, is of particular concern in PLWH since they have double the risk of developing CVD when compared to people not living with HIV (Barnes et al., 2017; Wagle et al., 2022; Perkins et al., 2023). Despite the fact that overall deaths in PLWH have declined over the years due to successful ART, deaths related to CVD have increased (Perkins et al., 2023).

**Figure 5:**
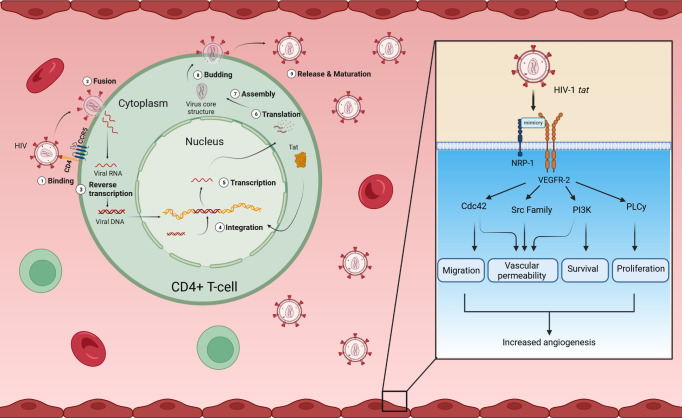
Intracellular and extracellular roles of HIV tat. HIV infects CD4+ T-cells. Intracellularly, HIV tat plays a role in the transcription of HIV genes. Extracellularly, HIV tat is a functional peptide mimetic for VEGF-A, binding to VEGFR-2 with high affinity and activating downstream signaling through its phosphorylation, similarly to the proper ligand, VEGF-A. The binding of HIV tat to VEGFR-2 can promote migration, vascular permeability, survival, and proliferation of endothelial cells, all factors that drive angiogenesis. (Created in Biorender). CCR5, C-C chemokine receptor type 5; CD4, cluster of differentiation 4; Cdc42, cell division control protein 42 homolog; NRP-1, neuropilin-1; PLC-γ, phospholipase C gamma; PI3K, phosphatidylinositol 3-kinase; Src, proto-oncogene tyrosine-protein kinase Src; Tat, trans-activator of transcription; VEGF-A, vascular endothelial growth factor A; VEGFR-2, vascular endothelial growth factor receptor 2.

The HIV-trans-activator of transcription (*tat*) protein has both an intracellular and extracellular role (Palakeel et al., 2022). Intracellularly, *tat* is essential for the transcription of HIV-1 in the host cell nucleus (Palakeel et al., 2022). Extracellular *tat* proteins, released from infected T cells, mimic VEGF-A by binding to VEGFR-2, thereby promoting angiogenesis via the activation of the fibroblast growth factor (FGF) and fetal liver kinase-1/kinase insert domain receptor (Flk-1/KDR) pathways, as well as PI3K/Akt, PLC-γ, Src/STAT3, and Rho GTPase cell division control protein 42 (Cdc42), all of which are involved in angiogenesis (**Figure 5**) (Arasteh and Hannah, 2000; Palakeel et al., 2022). *Tat* mimicking VEGF-A function has been implicated in the development of vascular tumors, such as Kaposi’s sarcoma in both mice and humans (Arasteh and Hannah, 2000). The protein can increase vascular endothelial cell proliferation by interacting with the alpha-v beta-3 integrin (αvβ3), thereby upregulating the NF-κB pathway (Palakeel et al., 2022).

Conversely, VEGF-A has also been shown to be neuroprotective against age-related cognitive decline in people without HIV (Hohman et al., 2015). A study done on Alzheimer’s disease found that increased concentrations of VEGF-A in the cerebrospinal fluid were associated with better brain aging, especially in individuals expressing early signs of the disease (Hohman et al., 2015). However, further studies are necessary to determine the exact mechanisms involved in neuroprotection. In PLWH, lower blood levels of VEGF-D (also an angiogenic protein) have been associated with higher odds of meeting the criteria for amnestic mild cognitive impairment (aMCI) status (Serrano et al., 2021). While this study did not find any association between blood concentrations of VEGF-A and aMCI in PLWH, it is possible that the balance between Ang-1 and Ang-2 might have an important role in this regard. Of interest are studies that have demonstrated that peroxisome proliferator-activated receptor-gamma (PPARγ) activation, reported to induce an increase in the Ang-1/Ang-2 ratio (Serghides et al., 2014), reduced HIV-1 or *tat*-induced dysfunction in micro-vessel endothelial cells in the brain (Huang et al., 2008; Huang et al., 2009).

A study by Korgaonkar et al. demonstrated that HIV infection increases the expression of VEGF-A through HIF-1α (Korgaonkar et al., 2008). A potential mechanism by which this may occur is through the *tat* protein, via attenuation of repair of damaged DNA by downregulating the DNA-protein kinase catalytic subunit (Palakeel et al., 2022). This, in turn, leads to the accumulation of ROS, which stimulate the release of HIF-1α (Palakeel et al., 2022).

Korgaonkar et al. also found that the HIV *nef* gene upregulates HIF-2α (rather than HIF-1α), which the authors suggest indicates a hypoxia-independent pathway (Korgaonkar et al., 2008). By activating the Src/STAT3 pathway, *nef* increases HIF-2α and VEGF-A in podocytes (Korgaonkar et al., 2008). They found that, at the mRNA level, VEGF-A isoforms 188 and 164 were upregulated in podocytes infected with HIV (Korgaonkar et al., 2008). Another significant finding is that, at both the RNA and protein level, the expression of VEGFRs (NRP-1, Flt1, and VEGFR-2) was upregulated in HIV-infected podocytes, which suggests that HIV could regulate the expression of VEGF-A receptors (Korgaonkar et al., 2008). The authors concluded that HIV induces upregulation of VEGFR-2 and NRP-1, which can facilitate and enhance the interaction of VEGF-A with its receptor, VEGFR-2 (Korgaonkar et al., 2008).

A recent study by Schnittmann et al. confirmed that NRP-1 has the same function in PLWH as in people without HIV (Schnittman et al., 2023). These include immune regulation, signal transduction, cell adhesion, and migration, as well as angiogenesis (Schnittman et al., 2023). The pathway was not related to major inflammatory pathways that are elevated in response to HIV infection (Schnittman et al., 2023). However, NRP-1 is upregulated on the surface of activated CD4+ T cells and is associated with T cell exhaustion; this was consistent with the association of elevated NRP-1 levels with a lower CD4+ T cell count (Schnittman et al., 2023). NRP-1 is also strongly associated with sCD163 in PLWH, which is an immunoregulatory marker that polarizes the monocyte/macrophage response toward an M2 phenotype (Schnittman et al., 2023). NRP-1 increases vascular permeability, which could be potentially detrimental under conditions of chronic inflammation induced by HIV infection (Schnittman et al., 2023).

NRP-1 expression is associated with multiple forms of cancer, as well as conditions such as type 2 myocardial infarction, in which there is an imbalance of oxygen supply and demand in the absence of an atherothrombotic coronary event (Schnittman et al., 2023). It has, therefore, been proposed that NRP-1 is a clinically significant immunoregulatory marker of co-morbidity in PLWH (Schnittman et al., 2023). The silencing of the *NRP1* gene has been shown to increase the infectivity of HIV by promoting the transmission of HIV in innate immune cells (dendritic cells and macrophages) (Naidoo et al., 2023). Further studies are needed to establish the role of NRP-1 in the development of comorbidities in PLWH.

Thakkar et al. studied the Ang-1 and Ang-2 levels in treated and untreated PLWH and compared both groups to uninfected healthy controls (Thakkar et al., 2021). They found that PLWH on ART had lower levels of Ang-1 and similar levels of Ang-2, whereas PLWH not on ART had similar levels of Ang-1 and higher levels of Ang-2, when compared to controls (Thakkar et al., 2021). Of note, PLWH not receiving ART had significantly higher levels of both Ang-1 and -2 when compared to PLWH on ART (Thakkar et al., 2021). Their results imply that both HIV infection and ART alter the ratio of Ang-1 and -2, which causes a disruption in endothelial and vascular homeostasis (Thakkar et al., 2021). They also demonstrated that HIV infection leads to endothelial dysfunction by impairing microvascular function, which in PLWH on ART reflects the toxicity of certain ART regimens and the impact of viral persistence on the microvasculature (Thakkar et al., 2021). The mechanisms behind the dysfunction caused by these factors require further research (Thakkar et al., 2021).

**Figure 6:**
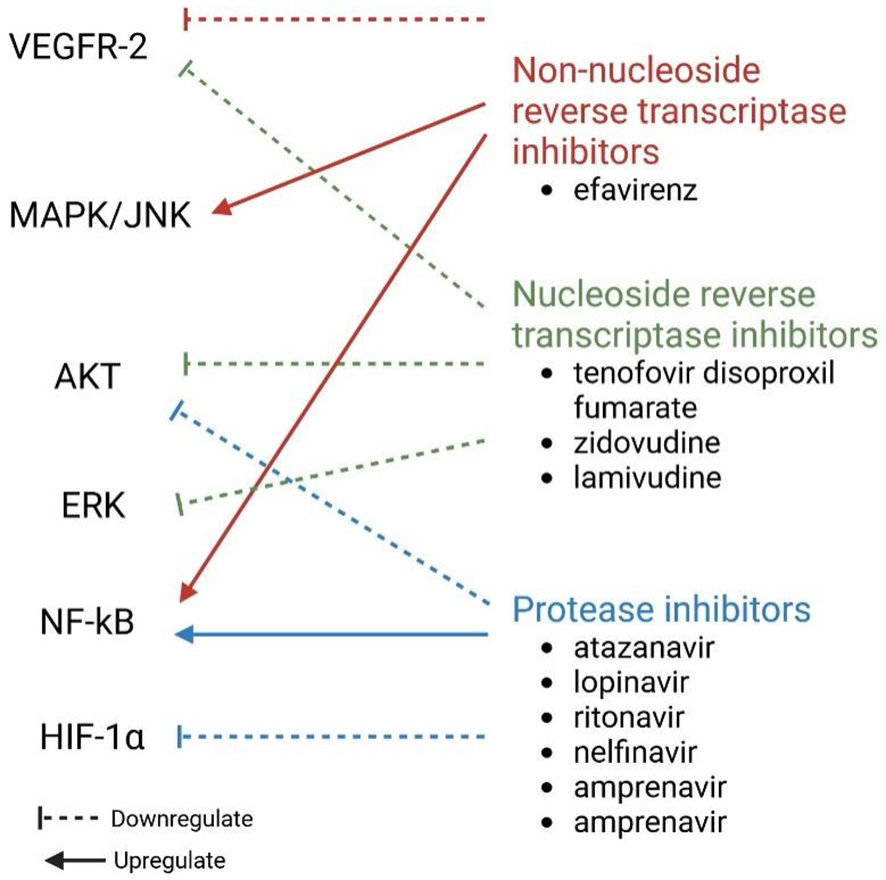
Effect of different ARTs on molecules involved in angiogenesis. ERK, Extracellular signal-regulated kinase; HIF-1α, hypoxia-inducible factor 1 subunit alpha; JNK, Jun N-terminal kinase; MAPK, mitogen-activated protein kinase; NF-κB, nuclear factor kappa light chain enhancer of activated B cells; VEGFR-2, vascular endothelial growth factor receptor 2.

As demonstrated in **Figure 6**, antiretroviral drugs can diversely affect angiogenesis (Barillari, 2020). Protease inhibitors, such as atazanavir, lopinavir, ritonavir, nelfinavir, amprenavir, and saquinavir can downregulate HIF-1α expression and activate the NF-κB pathway, which reduces VEGF-A production (Jamaluddin et al., 2010; Barillari, 2020). This is not true for indinavir (Dabravolski et al., 2022). Protease inhibitors can also decrease both total and phosphorylated Akt levels by reducing the levels of NO and eNOS produced by endothelial cells (Barillari, 2020), thus indicating that protease inhibitors can inhibit angiogenesis (Chang et al., 2018). Nucleos(t)ide reverse transcriptase inhibitors (N(t)RTIs), such as tenofovir disoproxil fumarate, zidovudine, and lamivudine, can block basic fibroblast growth factor and VEGFR-2 receptors on endothelial cells and weaken ERK and AKT phosphorylation by VEGF-A (Barillari, 2020). These changes lead to dysfunctional angiogenesis, which prohibits endothelial cell proliferation (Dunk and Serghides, 2022). Non-nucleoside reverse transcriptase inhibitors (NNRTIs), such as efavirenz, increase the activation of the NF-κB and MAPK/JNK pathways in endothelial cells (Jamaluddin et al., 2010; Barillari, 2020). Efavirenz has antiangiogenic activity in that it counteracts endothelial growth through the interaction of endothelial cell-generated NO and ROS caused by the drug (Argaw et al., 2009; Weiss et al., 2016; Barillari, 2020). The exact mechanism by which efavirenz induces ROS is still unknown. Song et al. showed that NNRTIs inhibit the VEGF-A/VEGFR-2 signaling pathway and the activity of receptor tyrosine kinases (RTKs) (Song et al., 2018). Efavirenz can also lead to increased vessel permeability by modifying endothelial cell junctions (Jamaluddin et al., 2010; Barillari, 2020).

### 6.3 SARS-CoV-2 and HIV co-infection

Hyperactivated platelets are a hallmark of both HIV- and SARS-CoV-2-infection (Venter et al., 2020; Ogweno, 2022). In individuals infected with HIV, platelet exhaustion due to persistent activation, despite virally suppressive ART, leads to reduced expression of granule-storing factors (Mesquita et al., 2018). Interestingly, Mesquita et al. hypothesized that increased platelet activation in PLWH may be a consequence of persistent microbial translocation (Mesquita et al., 2018). Recently, the integrity of the gut barrier of SARS-CoV-2-infected individuals has also been found to be compromised, as demonstrated by high plasma levels of markers of tight junction permeability, resulting in increased translocation of gut microbes and microbial products (Giron et al., 2021).

Similarly, platelets in SARS-CoV-2 infection are found to be hypo-responsive; however, in this instance, the platelets do not appear to be ‘exhausted’ and it is speculated that this may be a consequence of the platelets being sequestered to the endothelium (Schrottmaier et al., 2021). In the study by van der Mescht et al., lower levels of PDGF-BB and the chemokine, RANTES, both of which are produced by platelets and stored in the α-granules of these anucleate cells, were found in PLWH co-infected with SARS-CoV-2 (van der Mescht et al., 2023). In addition, the levels of TGF-β1, which, among other cell types, is also produced by platelets, were moderately lower in PLWH (van der Mescht et al., 2023). These results are consistent with platelet exhaustion. It should be noted, however, that lower PDGF-BB levels were associated with age and gender rather than co-infection (van der Mescht et al., 2023). This finding could suggest that the elevated levels of VEGF observed in PLWH co-infected with SARS-CoV-2 originate predominantly from activated immune cells because of the upregulation of HIF-1α by damaged endothelial cells, rather than release from the α-granules of platelets.

Endothelial dysfunction, as a consequence of HIV-induced systemic inflammation, is well documented (Kamtchum-Tatuene et al., 2019). Similarly, endothelial damage in individuals infected with SARS-CoV-2 is also well recognized (Otifi and Adiga, 2022; Xu et al., 2023). This is considered most likely due to the direct viral infection of endothelial cells, which express ACE-2 receptors, or to a host response to the infection (Varga et al., 2020). Connors et al. reported that the virus itself does not appear to have intrinsic procoagulant effects and that the coagulopathy observed in SARS-CoV-2 probably stems from the inflammatory response observed in infected individuals and the ensuing endothelial activation or damage (Connors and Levy, 2020). Although no results have yet been published, the ENDOCOVID project has set out to evaluate the long-term cardiovascular risk, including vascular endothelial function, in PLWH co-infected with SARS-CoV-2. This study will give valuable insight into identifying ‘an important transitional cardiovascular phenotype in COVID-19 (Goswami et al., 2021).

## 7 Anti-VEGF monoclonal antibodies as a treatment option for COVID-19

VEGF has been implicated as a good potential biomarker of SARS-CoV-2 infection and may be considered a target for therapy in acute infection. Bevacizumab is a recombinant monoclonal antibody that functions as an anti-VEGF agent (Lampropoulou et al., 2022). It inhibits angiogenesis by blocking the binding of VEGF-A to its receptors, VEGFR-1 and VEGFR-2 (Lampropoulou et al., 2022). It was initially approved by the FDA in 2004 for treating various cancers, including non-small-cell lung carcinoma, colorectal, kidney, cervical, and ovarian cancers, as well as glioblastoma (Chen K-H. et al., 2020; Lampropoulou et al., 2022). By reducing VEGF levels induced by hypoxia and severe inflammation and maintaining the integrity of the infected respiratory tract epithelium, bevacizumab, might help alleviate edema in patients with COVID-19 (Chen K-H. et al., 2020).

Bevacizumab’s clearance does not depend on renal or hepatic elimination (Lampropoulou et al., 2022). Instead, its metabolism follows endogenous IgG pathways, primarily through proteolytic catabolism (Lampropoulou et al., 2022). The long terminal half-life of the drug, approximately 20 days, is due to the binding of IgG to the neonatal Fc receptor (FcRn) (Lampropoulou et al., 2022). One advantage of this drug is that no dosage adjustments are necessary for patients with hepatic or renal impairment, as neither are involved in bevacizumab clearance (Lampropoulou et al., 2022). Consequently, pharmacokinetic drug-drug interactions (DDIs) are unlikely (Lampropoulou et al., 2022).

Drugs with secondary anti-VEGF properties include sunitinib and sorafenib, tyrosine kinase inhibitors that block both cytosolic VEGF and PDGF receptors (Sahebnasagh et al., 2021). However, there is limited data on their potential therapeutic impact in either COVID-19 or non-COVID-19-induced ARDS (Sahebnasagh et al., 2021). Other drugs with promising mechanisms of action but without data supporting their use in COVID-19 include the oral anti-angiogenesis inhibitor, rivoceranib, since it competitively and selectively inhibits VEGFR-2 and cyclosporine, an immunosuppressant with possible anti-VEGF effects, which also plays a vascular protective role (Sahebnasagh et al., 2021). At low concentrations, it exhibits anti-angiogenic and anti-apoptotic effects in endothelial cells (Sahebnasagh et al., 2021). Additionally, it has been reported that cyclosporine can downregulate VEGF through a cAMP-mediated signaling pathway in a dose-dependent manner (Sahebnasagh et al., 2021). Anti-VEGF drugs can either affect VEGF kinetics through direct VEGF inhibition, by blocking VEGFRs, or by directly inhibiting IL-6 with a secondary anti-VEGF impact (Sahebnasagh et al., 2021).

By utilizing a combination of search engines (PubMed and Google Scholar) and searching for key phrases, namely, “SARS-CoV-2”, “COVID-19 mortality”, “COVID-19 severity”, “anti-VEGF therapy” and “bevacizumab”, two studies (Islam et al., 2020; Valtuena et al., 2022), two published clinical trials (Pang et al., 2021; Blumberg et al., 2022), and three case reports (Patel et al., 2021; Fanning et al., 2022) were found describing anti-VEGF therapies that have been used to treat COVID-19.

It was interesting to note that a study conducted in Spain by Valtuena et al. reported that patients who received at least one intravitreal injection of anti-VEGF-A therapy (bevacizumab, ranibizumab, or aflibercept) for eye conditions between March and June 2020 had lower SARS-CoV-2 seroprevalence (0.86% versus 7.4%) and hospitalization (0.46% versus 4.1%) compared to the general population (Valtuena et al., 2022). These authors also stated that no deaths were reported in patients receiving anti-VEGF therapy (Valtuena et al., 2022). A single-arm trial (NCT04275414) conducted by Pang et al. involving 26 patients with severe COVID-19, undertaken between 15 February to 5 April 2020, administered a single dose of bevacizumab intravenously at 7.5 mg/kg and found that the treatment reduced the duration of oxygen therapy (Pang et al., 2021). The study reported significant improvement in the PaO2/FiO2 ratio on days 1 and 7, with 92% of patients showing improved oxygen-support status by day 28, and 65% being discharged (Pang et al., 2021). Chest imaging revealed a reduction in lesions within 7 days, and 93% of febrile patients had normalized body temperature within 72 hours (Pang et al., 2021). It is plausible that the effect of bevacizumab is related to the release of VEGF by leukocytes in response to the hypoxic environment and thus, by inhibiting VEGF it could remove the chemoattractant signal needed for the recruitment of monocytes and macrophages (Jaimes-Castelán et al., 2024). Safety data indicate that the most common adverse event was elevated hepatic enzymes, with other events including reduced hemoglobin and elevated blood pressure (Pang et al., 2021). The study suggests that bevacizumab plus standard care is highly beneficial for patients with severe COVID-19, warranting further randomized controlled trials to explore its global efficacy and safety (Pang et al., 2021).

In a phase 1 trial (NCT04412785) conducted by Blumberg et al., the safety and efficacy of cyclosporine A (CSA) in 10 hospitalized, oxygen-requiring, non-critically ill patients with COVID-19 were evaluated (Blumberg et al., 2022). Each patient received CSA at a dosage of 9 mg/kg/day (Blumberg et al., 2022). Clinical responses and adverse events were monitored, alongside serum cytokine and chemokine levels, and gene-expression analyses were also conducted (Blumberg et al., 2022). Adverse events were observed in five participants, with transaminitis being the most common; however, none were considered serious (Blumberg et al., 2022). Importantly, no patient required intensive care, and all patients were discharged (Blumberg et al., 2022). CSA administration was associated with significant reductions in serum cytokines and chemokines implicated in COVID-19 hyperinflammation, notably CXCL10 (Blumberg et al., 2022). Additionally, a marked decrease in type I IFN gene expression signatures and other hyperinflammatory transcriptional profiles was observed in peripheral blood post-treatment (Blumberg et al., 2022). The study concluded that CSA is a safe and potentially effective therapeutic intervention for SARS-CoV-2 infection (Blumberg et al., 2022). Its anti-inflammatory properties, widespread availability, safety profile, and low cost make it particularly advantageous for use in resource-limited settings (Blumberg et al., 2022). Based on these promising results, they proposed that further large-scale, randomized controlled trials are warranted to comprehensively assess the global safety and efficacy of CSA in the treatment of COVID-19 pneumonia (Blumberg et al., 2022).

In another study involving 199 patients with severe and critical COVID-19 admitted to the intensive care unit (ICU) of the Combined Military Hospital in Dhaka, Bangladesh, patients were treated with immune modulators: 102 patients received only one dose of tocilizumab (anti-IL-6), 48 received two doses of tocilizumab, 3 received three doses of tocilizumab, 25 patients received only one dose of bevacizumab, and the rest received a combination of the two monoclonal antibodies (Islam et al., 2020). The median lung involvement was 65%, and the mean oxygen saturation was 83% (Islam et al., 2020). The results showed a 71.5% survival rate for patients receiving a single dose of tocilizumab, while those treated with bevacizumab had a 92% survival rate (Islam et al., 2020). When both drugs were combined, the survival rate was 66% (Islam et al., 2020). The shortest ICU stay was observed in patients receiving a single dose of bevacizumab, whereas the longest stay was for those receiving tocilizumab (Islam et al., 2020). The study underscores the importance of early active treatment to prevent the progression of COVID-19 and highlights the safety of these immune modulators (Islam et al., 2020).

The first case report by Fanning et al., reported on a 52-year-old male with a history of hereditary hemorrhagic telangiectasia with severe epistaxis causing iron-deficient anemia, managed with iron infusions and blood transfusions over a 2 year period (Fanning et al., 2022). The patient’s epistaxis was controlled with six treatments of intravenous bevacizumab (5 mg/kg) from September to November 2021, raising his hemoglobin levels to 7.7 g/dL without requiring further iron treatment or transfusions (Fanning et al., 2022). In December 2021, 6 weeks post-treatment, he experienced severe epistaxis, leading to hospitalization (Fanning et al., 2022). On admission, his vitals were as follows: temperature 38.0°C (peak 39.4°C), heart rate 118 bpm, BP 119/64 mm Hg, respiratory rate 24 bpm, and saturation of peripheral oxygen (SpO2) of 99% on room air (Fanning et al., 2022). He tested positive for COVID-19 via a polymerase chain reaction (PCR) test (Fanning et al., 2022). The rest of his laboratory tests were normal (Fanning et al., 2022). During his 5-day hospital stay, he received iron infusions, 5 units of blood, and intravenous bevacizumab (5 mg/kg) and remdesivir on day 2 (Fanning et al., 2022). At discharge, his hemoglobin was 8.1 g/dL, and he was on room air (Fanning et al., 2022). He continues to be followed for hereditary hemorrhagic telangiectasia and receives bevacizumab every 2 weeks (Fanning et al., 2022). At the time of the report, he had no COVID-19-related sequelae (Fanning et al., 2022). Fanning et al. concluded that their report demonstrates the potential benefit of low-dose intravenous bevacizumab in a patient with moderate COVID-19 (Fanning et al., 2022).

Patel et al. published an article on two case reports of patients with COVID-19 who received anti-VEGF therapy (Patel et al., 2021). The first was of a 33-year-old female who presented with difficulty breathing, fever, and a dry cough and had a SARS-CoV-2 positive PCR test with a moderately high viral load (Patel et al., 2021). The patient also had diabetes and hypothyroidism (Patel et al., 2021). Her oxygen saturation was 87% and she was tachypneic and tachycardic when presenting to the emergency room (Patel et al., 2021). After treatment with remdesivir, corticosteroids, and supplemental oxygen (rebreather mask), the patient’s condition deteriorated and she was moved to non-invasive bi-level positive airway pressure ventilation support (Patel et al., 2021). On the second day, the patient’s ratio of partial pressure of oxygen to the fraction of inspired oxygen (PaO2/FiO2) was 118 and she received a standard dose of bevacizumab (Patel et al., 2021). Over the next few days, her clinical symptoms improved (Patel et al., 2021). On day 7, her PaO2/FiO2 was 290 and her chest x-ray indicated improvement of the lung ground-glass opacity (Patel et al., 2021).

The second case report was of a 30-year-old male who presented with fever and difficulty breathing with a SARS-CoV-2 positive PCR test and a high viral load (Patel et al., 2021). On presentation to the emergency room, his oxygen saturation was 80%, he was tachycardic and tachypneic and received oxygen support via a non-rebreather mask (Patel et al., 2021). The patient’s PaO2/FiO2 was 89 and the patient received non-invasive bi-level positive airway pressure ventilation support (Patel et al., 2021). Remdesivir, corticosteroids, and a standard dose of bevacizumab were administered (Patel et al., 2021). Over the next few days, his chest x-ray showed improvement and his PaO2/FiO2 increased to 240 (Patel et al., 2021).

Improvements in both patients were seen 24 hours after the administration of bevacizumab (Patel et al., 2021). Seven days following admission, the PaO2/FiO2 and chest X-ray imaging showed improvement in both patients (Patel et al., 2021). The first patient was discharged 15 days after admission and the second patient 23 days after admission (Patel et al., 2021).

Ongoing clinical trials are investigating the use of the following anti-VEGF agents for the treatment of severe COVID-19: i) a phase 2 study investigating the efficacy and safety of siltuximab (NCT04329650) (ClinicalTrials.gov, 2024a), ii) a phase 3 study investigating the time to recovery from a category 0 to 5 on the WHO progression scale (NCT04822818) following bevacizumab administration (ClinicalTrials.gov, 2024b), iii) a single-center pilot study in Mexico comparing treatment with steroids and CSA or steroids alone, with the primary outcome being the number of days to clinical improvement, until hospital discharge or death (NCT04540926) (ClinicalTrials.gov, 2024c), iv) a phase 2 randomized clinical trial of non-ICU, but hospitalized patients, receiving CSA 2.5 mg/kg and standard care (NCT04492891) (ClinicalTrials.gov, 2024d), and v) a randomized control trial investigating the efficacy of CSA in improving the prognosis of hospitalized patients (NCT04392531) (ClinicalTrials.gov, 2024e).

On the other hand, while normal vasculature does not rely on VEGF for survival and function, angiogenesis is crucial for wound healing. Agents affecting vascular growth can therefore disrupt this process (Sahebnasagh et al., 2021). VEGF is vital for the normal function of organs such as the liver, kidneys, neurons, and blood vessels, thus inhibiting it can lead to clinical complications (Sahebnasagh et al., 2021). Preclinical studies show that systemic administration of anti-VEGF drugs for 1-3 weeks can cause reversible vascular regression (Sahebnasagh et al., 2021). Hypertension has been reported to occur in up to 32% of patients, likely due to reduced NO production or VEGF inhibition in the kidneys (Sahebnasagh et al., 2021). Nephrotic syndrome and proteinuria, seen in up to 23% of patients, are usually asymptomatic and resolve after stopping treatment (Sahebnasagh et al., 2021). Gastrointestinal perforation, though rare, can be life-threatening, with a 1.5% occurrence in colorectal cancer patients treated with bevacizumab (Sahebnasagh et al., 2021).

Other reported complications include gastrointestinal, cerebral, and vaginal bleeding; thromboembolism; myocardial infarction; and angina, with higher risks in older patients or those with a history of these events (Sahebnasagh et al., 2021). Venous and arterial thrombosis may result from VEGF inhibition due to its effect on endothelial cell survival and vascular integrity (Sahebnasagh et al., 2021). Cardiac complications, such as reduced function and myocardial ischemia, have been noted with sunitinib and sorafenib (Sahebnasagh et al., 2021). Reversible leukoencephalopathy, cerebral edema, and blood-brain barrier disruption are also potential side effects (Sahebnasagh et al., 2021). Additionally, increased blood thyroid-stimulating hormone levels and thyroid dysfunction have been observed, potentially causing fatigue (Sahebnasagh et al., 2021).

A study that administered bevacizumab to patients hospitalized with COVID-19 not only found no significant benefit of the drug, but one patient also developed spontaneous hemoptysis as a complication of anti-VEGF therapy (Marwah et al., 2021). Complete inhibition of the binding of VEGF to its receptors could cause alveolar septal cell apoptosis due to its antiapoptotic function in endothelial cells (Rajachandran et al., 2022). Another factor that needs to be considered is genetic differences in terms of VEGF polymorphisms, which could mean variations in VEGF plasma levels as well as alterations in pathways between individuals (Rajachandran et al., 2022). Some of the genetic variants of VEGF are known to predispose individuals to develop ARDS or acute lung injury (Rajachandran et al., 2022). The possible side effects related to anti-VEGF treatment indicate the need for treatment that selectively blocks the interaction of VEGF with VEGFR-1, which has been implicated in COVID-19 pathogenesis. Currently, a few experimental monoclonal antibodies (KM1730/KM1732, D16F7, and IMC-18F1/icrucumab) with VEGFR-1 specificity are in development (Ceci et al., 2020). Of these, only IMC-18F1/icrucumab has progressed to a phase 2 clinical trial where it was used in combination with modified FOLFOX-6 in patients with metastatic colorectal cancer. Unfortunately, this combination did not improve progression-free survival (Moore et al., 2016).

Adverse effects have also been reported for icrucumab and other VEGFR-1 inhibitors, such as cabozantinib. A phase I clinical trial enrolled patients with advanced solid malignancies with the primary objective of determining the safety profile and maximum tolerated dose of icrucumab in found that two out of the 27 patients enrolled developed severe side effects (LoRusso et al., 2014). The first experienced hyponatremia and dehydration and the second developed Grade 3 anaemia (LoRusso et al., 2014). These adverse events were possibly related to icrucumab and resolved after the patients halted treatment (LoRusso et al., 2014). The most common adverse events in the study included fatigue (50%), nausea (46.2%), peripheral edema (46.2%), anemia (42.3%), and dyspnea (42.3%) (LoRusso et al., 2014). Hypertension was not reported (LoRusso et al., 2014). Cardiovascular events were rare, with only three cases of tachycardia reported, none of which were linked to the study drug (LoRusso et al., 2014). Among the 11 patients with anemia, six had pre-existing mild to moderate anemia at baseline (LoRusso et al., 2014). During the study, hemoglobin levels increased in 88.5% of patients (LoRusso et al., 2014). A systemic literature review done by Maroto et al. which included 114 studies done regarding the safely and efficacy of Cabozantinib in the treatment of solid tumors found that adverse events were consistent across different cancer types and similar to those seen with other tyrosine kinase inhibitors (TKIs) (Maroto et al., 2022). The most common adverse events included hypertension, diarrhea, fatigue, elevated lipase levels, and palmar-plantar erythrodysesthesia syndrome (PPES) (Maroto et al., 2022). Hyponatremia was more frequent at higher doses of cabozantinib (140 mg compared to 60 mg) (Maroto et al., 2022). These adverse events were effectively managed through dose adjustments (Maroto et al., 2022).

The aforementioned experimental monoclonal antibodies have not been studied in the context of COVID-19, thus, further clinical trials are needed to test the efficacy and safety of VEGFR-1 inhibitors for the treatment of COVID-19. Furthermore, to our knowledge, no studies have evaluated the safety and efficacy of anti-VEGF therapy in PLWH who have contracted SARS-CoV-2, highlighting the need for further research.

## 8 Conclusions

In this review article, the molecular interactions of VEGF-A and its receptors, VEGFR-1 and VEGFR-2, and co-receptor NRP-1, as well as their roles in the pathogenesis of SARS-CoV-2 and HIV infection have been addressed. Both infections are shown to cause vascular endothelium dysfunction through the disruption of vascular homeostasis and angiogenesis. VEGF-A signaling can result in either sprouting or intussusceptive angiogenesis, depending on the local concentration of this proangiogenic cytokine, the location, and the underlying pathological processes. The release of VEGF-A from smooth muscle cells and immune cells, such as macrophages, culminates in generalized vascular activation, proliferation, and increased permeability. This, in turn, promotes endothelial cell migration towards hypoxic areas where these cells assist in forming new blood vessels to compensate for ischemia and damage secondary to hypoxia. In SARS-CoV-2 infection, the virus binds to NRP-1, diverting the free VEGF-A to bind to soluble VEGFR-1 and VEGFR-1 expressed on the surface of immune cells. This interrupts endothelial repair and increases inflammation. Higher levels of VEGF-A increase vascular permeability and ultimately lead to pulmonary edema. The HIV *tat* protein also mimics VEGF-A and HIV infection also upregulates NRP-1. Both of these factors augment angiogenesis in HIV infection. In the case of SARS-CoV-2/HIV co-infection, we speculate that the elevated levels of VEGF-A observed in this clinical setting originate predominantly from activated immune cells because of upregulation of HIF-1α by damaged endothelial cells. It has been shown that anti-VEGF treatment could potentially improve COVID-19 disease progression, with the caveat that completely blocking VEGF can have negative effects, which may disrupt physiological angiogenesis. Thus, although treatments designed to target VEGFR-1 specifically could be beneficial in the context of SARS-CoV-2 infection, further research is necessary to provide additional insights into the benefits of anti-VEGF therapy in SARS-CoV-2 infection, and especially in SARS-CoV-2/HIV co-infection.
